# Key interactions by conserved polar amino acids located at the transmembrane helical boundaries in Class B GPCRs modulate activation, effector specificity and biased signalling in the glucagon-like peptide-1 receptor

**DOI:** 10.1016/j.bcp.2016.08.015

**Published:** 2016-10-15

**Authors:** Denise Wootten, Christopher A. Reynolds, Kevin J. Smith, Juan C. Mobarec, Sebastian G.B. Furness, Laurence J. Miller, Arthur Christopoulos, Patrick M. Sexton

**Affiliations:** a Drug Discovery Biology, Monash Institute of Pharmaceutical Sciences and Department of Pharmacology, Monash University, Parkville, Victoria 3052, Australia; b School of Biological Sciences, University of Essex, Wivenhoe Park, Colchester CO4 3SQ, UK; c Department of Molecular Pharmacology and Experimental Therapeutics, Mayo Clinic, Scottsdale, AZ 85259, USA

**Keywords:** cAMP, 3′,5′-cyclic adenosine monophosphate, CHO, Chinese hamster ovary, CRF1R, corticotrophin releasing factor receptor-1, DMEM, Dulbecco’s modified Eagle medium, FBS, fetal bovine serum, GCGR, glucagon receptor, GLP-1, glucagon-like peptide-1, GPCR, G protein-coupled receptor, _i_Ca^2+^, intracellular calcium, pERK, extracellular signal-regulated kinase 1 and 2 phosphorylation, PBS, phosphate buffered saline, TM, transmembrane helix, Glucagon-like peptide-1 receptor, Biased agonism, G protein-coupled receptor, Cell signaling

## Abstract

Class B GPCRs can activate multiple signalling effectors with the potential to exhibit biased agonism in response to ligand stimulation. Previously, we highlighted key TM domain polar amino acids that were crucial for the function of the GLP-1 receptor, a key therapeutic target for diabetes and obesity. Using a combination of mutagenesis, pharmacological characterisation, mathematical and computational molecular modelling, this study identifies additional highly conserved polar residues located towards the TM helical boundaries of Class B GPCRs that are important for GLP-1 receptor stability and/or controlling signalling specificity and biased agonism. This includes (i) three positively charged residues (R3.30^227^, K4.64^288^, R5.40^310^) located at the extracellular boundaries of TMs 3, 4 and 5 that are predicted in molecular models to stabilise extracellular loop 2, a crucial domain for ligand affinity and receptor activation; (ii) a predicted hydrogen bond network between residues located in TMs 2 (R2.46^176^), 6 (R6.37^348^) and 7 (N7.61^406^ and E7.63^408^) at the cytoplasmic face of the receptor that is important for stabilising the inactive receptor and directing signalling specificity, (iii) residues at the bottom of TM 5 (R5.56^326^) and TM6 (K6.35^346^ and K6.40^351^) that are crucial for receptor activation and downstream signalling; (iv) residues predicted to be involved in stabilisation of TM4 (N2.52^182^ and Y3.52^250^) that also influence cell signalling. Collectively, this work expands our understanding of peptide-mediated signalling by the GLP-1 receptor.

## Graphical abstract

**Figure:**
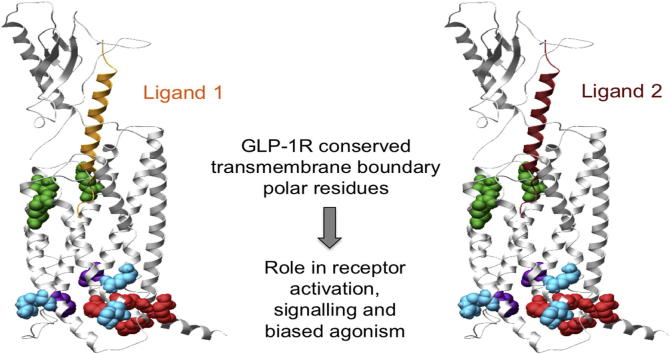

## 1 Introduction

GPCRs mediate signal transduction across cell membranes in response to a wide range of extracellular stimuli [43]. Understanding how these receptors function at the molecular level requires knowledge of how agonist binding is converted to receptor activation and consequently stimulation of downstream signalling cascades that can be both G protein-dependent and G protein-independent [37]. GPCRs are dynamic proteins that can explore multiple conformational states and with the advances in GPCR structural biology, new insights into the structural basis of GPCR activation have revealed the importance of inter-connected networks of residues for conformational transitions that allow agonist bound receptors to activate intracellular signalling cascades [29], [40].

Sequence alignments of related membrane proteins suggest that polar residues are under evolutionary pressure for conservation and hence maintain common structural and functional roles [25], [26]. In support of this, there are a number of highly conserved polar residues present in Class A GPCRs that participate in key interactions associated with their activation [4], [5], [42]. Class B GPCRs typically contain more conserved polar residues in their transmembrane (TM) bundle than Class A GPCRs, which may be reflective of the diversity of receptors/ligands found within the Class A subfamily, however, it may also reflect the mode by which Class B ligands bind and activate their receptors. Peptide ligands associate primarily with the large extracellular N-terminal domain of Class B GPCRs, but also need to interact with the TM bundle to promote receptor activation [6], [47], [48], [41]. Previously, we revealed the importance of networks of conserved polar residues located in the TM bundle of Class B GPCRs for controlling receptor activation and downstream signalling of the glucagon-like peptide-1 receptor (GLP-1R) [64], [66], [68]. This receptor plays an essential role in nutrient regulated insulin release, and has emerged as a major target for therapeutic treatment of type 2 diabetes and obesity. The GLP-1R is pleiotropically coupled to multiple signalling pathways with evidence for biased agonism by the physiological ligand oxyntomodulin, clinically used peptide mimetics and synthetic non-peptide ligands, relative to the cognate agonist GLP-1 [33], [65], [67]. In our previous studies, we identified conserved buried polar residues were not only important in receptor activation, but that some of these residues were also important for biased agonism at this receptor. The breakthrough crystal structures of the inactive TM domain of two Class B GPCRs (the glucagon receptor (GCGR) and the corticotrophin releasing factor receptor-1 (CRF1R)) that were subsequently published, largely supported the predictions and conclusions from the molecular modelling in these studies, highlighting that these conserved residues may form conserved hydrogen bond networks that are important for activation transition of all members of this class of GPCRs [22], [49].

**Fig. 1:**
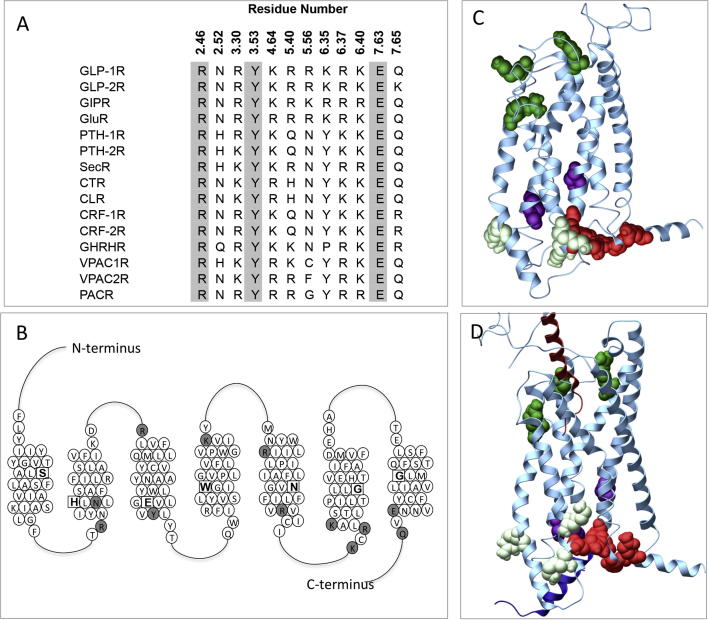
Conservation and location of polar residues mutated in this study. (A) Conservation of polar residues mutated in this study across the human Class B GPCRs (the secretin-like subclass). Residues absolutely conserved are highlighted in grey. These residues shown are conserved as polar (with the exception of 5.56 and 6.35 where one receptor subtype is not) across all mammalian species of receptor cloned to date. GLP-1R; glucagon-like peptide-1 receptor, GLP-2R; GLP-2 receptor, GIP, gastric inhibitory polypeptide receptor; GluR, glucagon receptor; PTH-1R, parathyroid hormone receptor 1; PTH-2R, PTH receptor 2; SecR, secretin receptor; CTR, calcitonin receptor; CLR, calcitonin-like receptor; CRF1, corticotropin-releasing factor receptor 1; CRF2, corticotropin-releasing factor receptor 2; GHRHR, GH-releasing hormone receptor; VPAC1R, vasoactive intestinal polypeptide type-1 receptor; VPAC2R, vasoactive intestinal polypeptide type-2 receptor, PACR, pituitary adenylate cyclase activating polypeptide 1 receptor. (B) Schematic representation of the TM domain of the human GLP-1R. The most conserved residue in each helix is highlighted as a square with a bold letter and represent residue 0.50 for that helix. Residues mutated in the present study are shown in grey. (C) Three-dimensional molecular homology model of the inactive TM bundle of the GLP-1R. (D) Three-dimensional molecular model of the TM bundle of the active full length model of the GLP-1R. The bound GLP-1 peptide is shown dipping into the bundle (dark red helix) and the G_αs_ peptide fragment bound at the intracellular face is shown in dark blue. In (C) and (D), side chains mutated in this study are highlighted in space fill with dark green indicating positively charged residues located towards the extracellular face of the bundle and interact with ECL2; pale green, positively charged residues located towards the intracellular face that may interact with lipid headgroups; red, residues in TMs 2, 6 and 7 that form a hydrogen bond network in the apo receptor; purple, residues in TMs 2 and 3 that stabilise interactions with TM4. (For interpretation of the references to colour in this figure legend, the reader is referred to the web version of this article.)

The high resolution TM domain structures have provided better structural templates for Class B GPCR modelling and enabled the generation of a homology model of the inactive state of the GLP-1R TM bundle [64], [68]. In addition to the hydrogen bond networks predicted in our previous model, the new model identified another network of residues in the inactive GLP-1R. This was formed between conserved Class B polar residues located within TMs 2, 6 and 7 at the intracellular face of the receptor and was also evident in the crystal structures of the GCGR and the CRF1R [22], [49]. In addition to participation in hydrogen bond networks, polar side chains located within the TM bundle of GPCRs can have other important functions. These include the formation of interactions with ligands or effectors and their ability to snorkel out towards phospholipid head groups, thereby stabilising TM helices within the bilayer [51]. These functions of polar TM residues are often (although not always) limited to residues that reside either towards the extracellular or intracellular TM boundaries. While our earlier studies on the GLP-1R focused on conserved polar residues that our original model predicted to reside in water-mediated hydrogen bond interaction networks, or in the central region of the TM bundle forming helical packing interactions, this current study explores the roles of the remaining conserved Class B GPCR TM polar residues, which are predicted to reside close to the TM boundaries (Fig. 1). This set of residues includes the amino acids located within the additional hydrogen bond network at the intracellular face of Class B GPCRs. We have assessed the role of these residues on GLP-1R function using a combination of mutagenesis, molecular modelling and pharmacological characterisation of multiple ligands for affinity and activation of three signalling endpoints. This identified residues important for ligand affinity, receptor folding and those contributing to biased agonism, expanding the current understanding of the functional role of highly conserved polar residues within Class B GPCRs.

## 2 Materials and methods

### 2.1 Materials

Dulbecco’s modified Eagle’s medium (DMEM), hygromycin-B and Fluo-4 acetoxymethyl (AM) ester were purchased from Invitrogen (Carlsbad, CA, USA). Foetal bovine serum (FBS) was purchased from Thermo Fisher Scientific (Melbourne, VIC, Australia). The QuikChange™ site-directed mutagenesis kit was purchased from Stratagene (La Jolla, CA, USA). AlphaScreen™ reagents, Bolton-Hunter reagent [^125^I] and 384-well ProxiPlates were purchased from PerkinElmer Life and Analytical Sciences (Waltham, MA, USA). SureFire™ ERK1/2 reagents were generously supplied by TGR Biosciences (Adelaide, SA, Australia). SigmaFast o-phenylenediamine dihydrochloride (OPD) tablets and antibodies were purchased from Sigma–Aldrich (St. Louis, MO, USA). GLP-1 peptides were purchased from Mimotopes (Clayton, VIC, Australia). All other reagents were purchased from Sigma–Aldrich (St. Louis, MO, USA) or BDH Merck (Melbourne, VIC, Australia) and were of an analytical grade.

### 2.2 Residue numbering

Throughout, residues were numbered using the numbering system described previously [66], whereby the most conserved residue in each Class B GPCR TM domain was assigned 0.50 with this number preceded by the TM number. Each residue is numbered according to its relative position to the residue at 0.50 in each helix and its absolute residue number is shown in superscript. The relative positions of the residues assessed in this study are shown in Fig. 1B–D.

### 2.3 Receptor mutagenesis

To study the influence of polar TM amino acids on receptor function, the desired mutations were introduced to an N-terminally double c-myc labelled wildtype human GLP-1R in the pEF5/FRT/V5-DEST destination vector (Invitrogen); this receptor had equivalent pharmacology to the untagged human GLP-1R. Mutagenesis was carried out using oligonucleotides for site-directed mutagenesis purchased from GeneWorks (Hindmarsh, SA, Australia) and the QuikChange™ site-directed mutagenesis kit (Stratagene). Sequences of receptor clones were confirmed by automated sequencing at the Australian Genome Research Facility. Mutated residues and their conservation across human Class B peptide hormone receptors are illustrated in Fig. 1.

### 2.4 Transfections and cell culture

Wildtype and mutant human GLP-1R were isogenically integrated into FlpIn-Chinese hamster ovary (FlpInCHO) cells (Invitrogen) and selection of receptor-expressing cells was achieved through treatment with 600 μg ml^−1^ hygromycin-B. Transfected and parental FlpInCHO cells were maintained in DMEM supplemented with 10% heat-inactivated FBS and incubated in a humidified environment at 37 °C in 5% CO_2_. For all experiments cells passages 8–20 were used.

#### 2.4.1 Radioligand binding assay

FlpInCHO wildtype and mutant human GLP-1R cells were seeded at a density of 3 × 10^4^ cells/well into 96-well culture plates and incubated overnight at 37 °C in 5% CO_2_, and radioligand binding carried out as previously described [32]. Briefly, binding assays were performed on whole cells incubated overnight at 4 °C with 0.05 nM ^125^I-exendin-4(9–39) tracer and increasing concentrations of unlabelled peptide. Cells were washed, solubilised in 0.1 M NaOH and radioactivity determined by γ-counting. For each cell line in all experiments, total binding was defined by 0.05 nM ^125^I-exendin-4(9–39) alone, and nonspecific binding was defined by co-incubation with 1 μM exendin-4(9–39). For analysis, data are normalised to the specific binding for each individual experiment.

### 2.5 cAMP accumulation assay

FlpInCHO wildtype and mutant human GLP-1R cells were seeded at a density of 3 × 10^4^ cells/well into 96-well culture plates and incubated overnight at 37 °C in 5% CO_2_. cAMP assays were carried out as previously described [33]. Briefly, cells were incubated with increasing concentrations of peptide ligands for 30 min at 37 °C in the presence of IBMX. Cells were lysed and cAMP levels were detected using a cAMP AlphaScreen™ detection kit (PerkinElmer). All values were converted to concentration of cAMP using a cAMP standard curve performed in parallel, and data were subsequently normalised to the response of 100 μM forskolin in each cell line.

### 2.6 pERK1/2 assay

FlpInCHO wildtype and mutant human GLP-1R cells were seeded at a density of 3 × 10^4^ cells/well into 96-well culture plates and incubated overnight at 37 °C in 5% CO_2_. Ligand-mediated pERK1/2 was determined using the AlphaScreen™ ERK1/2 SureFire™ protocol as previously described [39]. Briefly, cells were serum starved for 6 h prior to assay. Initial pERK1/2 time course experiments were performed over 1 h in the presence of either vehicle or 1 μM peptide to determine the time at which agonist-mediated pERK1/2 was maximal. pERK1/2 was detected using the AlphaScreen™ ERK1/2 SureFire™ kit. Subsequent experiments were then performed with increasing concentrations of peptides at the time required to generate a maximal pERK1/2 response using 1 μM peptide. The kinetics of pERK1/2 response for each mutant receptor was similar to WT, peaking at 6 min. Data were normalised to the maximal response elicited by 10% FBS in each cell line, determined at 6 min (peak FBS response).

### 2.7 _i_Ca^2+^ mobilisation assay

FlpInCHO wildtype and mutant human GLP-1R cells were seeded at a density of 3 × 10^4^ cells/well into 96-well culture plates and incubated overnight at 37 °C in 5% CO_2_, and receptor-mediated _i_Ca^2+^ mobilisation determined as previously described [61]. Briefly, cells were incubated for 1 h with the cell-permeant Ca^2+^ fluorophore, Fluo-4/AM (10 μM) in the presence of 2 mM probenecid prior to determining peptide-mediated changes in fluorescence in a Molecular Devices FlexStation (Molecular Devices, Palo Alto, CA, USA). Fluorescence was determined immediately after peptide addition, with an excitation wavelength set to 485 nm and an emission wavelength set to 520 nm, and readings taken every 1.36 s for 120 s. Peak magnitude was calculated using five-point smoothing, followed by correction against basal fluorescence. The kinetics for ligand-mediated _i_Ca^2+^ were not altered by any of the mutations. The peak value was used to create concentration–response curves. Data were normalised to the maximal response elicited by 100 μM ATP.

### 2.8 Cell surface receptor expression

FlpInCHO wildtype and mutant human GLP-1R cells, with receptor DNA previously incorporated with an N-terminal double c-myc epitope label, were seeded at a density of 25 × 10^4^ cells/well into 24-well culture plates and incubated overnight at 37 °C in 5% CO_2_, washed three times in 1× PBS and fixed with 3.7% paraformaldehyde (PFA) at 4 °C for 15 min. Cell surface receptor detection was then performed using a cell surface ELISA protocol to detect the cMyc epitope tag located at the extracellular N-terminus of the receptor, as previously described [32]. Data were normalised to the basal fluorescence detected in FlpInCHO parental cells. Specific ^125^I-exendin-4(9–39) binding at each receptor mutant, as identification of functional receptors at the cell surface, was also determined (corrected for nonspecific binding using 1 μM exendin-4(9–39)) as described in [65].

### 2.9 Molecular modelling

Two GLP-1R models were used to aid interpretation of mutational data; the methods for generation of these models have been described previously [64]. Briefly, the molecular models were constructed in three stages. An NMR structural ensemble of a short, conformationally constrained GLP-1 agonist (equivalent to GLP-1(7–18), pdb code 2N0I [21], was docked into a preliminary TM comparative model of GLP-1R, which was based on the glucagon X-ray crystal structure (PDB code 4L6R, (Sui et al., 2013)), using Glide (v6.9) SP peptide and the OPLS force field [56]. The conformationally constrained peptide was mutated to GLP-1 using PLOP [27]. GLP-1(7–18) was structurally aligned with GLP-1(10–35) co-crystallised with the ECD (PDB code 3IOL [58]), using VMD [24]. Duplicated residues were selectively removed from the complex, thus creating two overlapping templates that were key to combining the TM and ECD domains. These templates and the relevant portions of the X-ray structure of the β_2_-adrenergic receptor: G protein complex [45] were used to generate 2000 full length active GLP-1R (R27-R421) models containing the GLP-1(7–36)-NH_2_ peptide and the C-terminal peptide of the G protein (Gs) (R374-L394) using the comparative modelling programme Modeller 9.16 [16]; the modelling was carried out in the presence of a set of distance constraints as described in [64]. These structures are available from ftp://ftp.essex.ac.uk/pub/oyster/Wootten_JBC_2016/ (username ftp, password anonymous).

### 2.10 Molecular dynamics simulations

The GLP-1R model was inserted into a hydrated equilibrated palmitoyloleoylphosphatidylcholine (POPC) bilayer using the CHARMM-GUI interface [28]. Potassium and chloride ions were added to neutralise the system at an ionic strength of approximately 150 mM. Lipid14 (for POPC), AMBER99SP (for the protein) and TIP3P water model parameters were added using ambertools [7]. The simulations were carried out using ACEMD [19] on a purpose-built metrocubo GPU workstation. The system was energy minimised, heated from 0 K to 300 K in the NVT ensemble for 160 ps then simulated in the NPT ensemble, with 10 kcal mol^−1^ A^−2^ positional harmonic restraints applied to the protein heavy atoms, which were progressively reduced to 0 over the course of 15 ns. Bond lengths to hydrogen atoms were constrained using M-SHAKE [34]. Production simulations were performed in the NPT ensemble at 300 K and 1 atm, using a Langevin thermostat for temperature coupling and a Berendsen barostat for pressure coupling. Non-bonded interactions were cutoff at 10.0 Å, and long-range electrostatic interactions were computed using the particle mesh Ewald method (PME) with dimensions of 86×86×142 using a spacing of 1.00 Å. The unconstrained simulation was run for 500 ns. Quantitative analysis of the trajectory was conducted in VMD.

### 2.11 Data analysis

All data were analysed using Prism 6 (GraphPad Software Inc., San Diego, CA, USA). For all analyses the data are unweighted and each y value (mean of replicates for each individual experiment) is considered an individual point. To calculate IC_50_, EC_50_ and E_max_ values, concentration response signalling data were analysed as previously described [30] using a three-parameter logistic equation. IC_50_ values obtained from binding studies were then corrected for radioligand occupancy as previously described using the radioligand affinity (K_i_) experimentally determined for each mutant.

To quantify efficacy in the system, all data were fitted with an operational model of agonism to calculate estimated τ values. τ is the operational measure of efficacy in the system, which incorporates signalling efficacy and receptor density. This model has been extensively described previously [30], [66], [64]. All estimated τ values were then corrected to cell surface expression (τ_c_) as determined by cell surface ELISA and errors propagated from both τ and cell surface expression.

Signalling bias was also quantified as previously described by analysis of concentration–response curves with nonlinear regression using an operational model of agonism, but modified to directly estimate the ratio of τ_c_/K_A_ [30], [66], [64]. All estimated τ_c_/K_A_ ratios included propagation of error for both τ_c_ and K_A_. Changes in τ_c_/K_A_ ratios with respect to wildtype of each mutant were used to quantitate bias between signalling pathways. Accordingly, bias factors included propagation of error from τ_c_/K_A_ ratios of each pathway.

### 2.12 Statistics

Changes in peptide affinity, potency, efficacy, cell surface expression and bias of each mutant receptor in comparison to the wildtype control were statistically analysed with one-way analysis of variance and Dunnett’s post test, and significance was accepted at p < 0.05.

## 3 Results

Sequence alignments of the human Class B receptor subtypes reveal 22 conserved polar residues that are predicted to reside either in the TM bundle or at the membrane interface (10 of which are absolutely conserved as the same residue). An additional 2 residues are also very highly conserved in this subfamily (with the exception of 1 receptor subtype for loci 6.35 and 3 receptor subtypes for 5.56). We have previously reported the effects of mutation of 13 of these residues in the GLP-1R [64], [66]. In this study we have probed the function of the remaining residues (Fig. 1A). All of these are located at TM helical boundaries/interfaces with loops, with the exception of Q7.65^410^ that is located intracellularly within the predicted helix 8 (H8) at the bottom of TM7 (Fig. 1B–D). Each residue was individually mutated to Ala, verified by DNA sequencing and analysed for the effect of mutation on receptor function.

Wildtype and mutant human GLP-1Rs were isogenically integrated into FlpInCHO host cells by recombination that allows for direct comparison of cell surface expression as there should not be variations that arise due to differences in gene transcription. Cell surface expression was assessed by both antibody detection of the N-terminal double c-myc epitope label using ELISA and whole cell binding using [^125^-I]-exendin-4(9–39) (Table 1). A number of mutations resulted in significantly altered cell surface expression relative to the wildtype receptor, with consistent expression changes observed using both methods. Whole cell equilibrium competition binding studies were used to assess orthosteric peptide ligand affinities for the wildtype and each of the mutant GLP-1Rs (Table 1). These were performed with the endogenous agonists GLP-1(7–36)NH_2_ (GLP-1) and oxyntomodulin, in addition to the exogenous agonist exendin-4 and an antagonist exendin-4(9–39), all in competition with the radiolabelled ligand ^125^I-exendin-4(9–39). This revealed a number of mutations that globally altered peptide affinity and those that had selective effects of peptide affinity (Table 1).

**Fig. 2:**
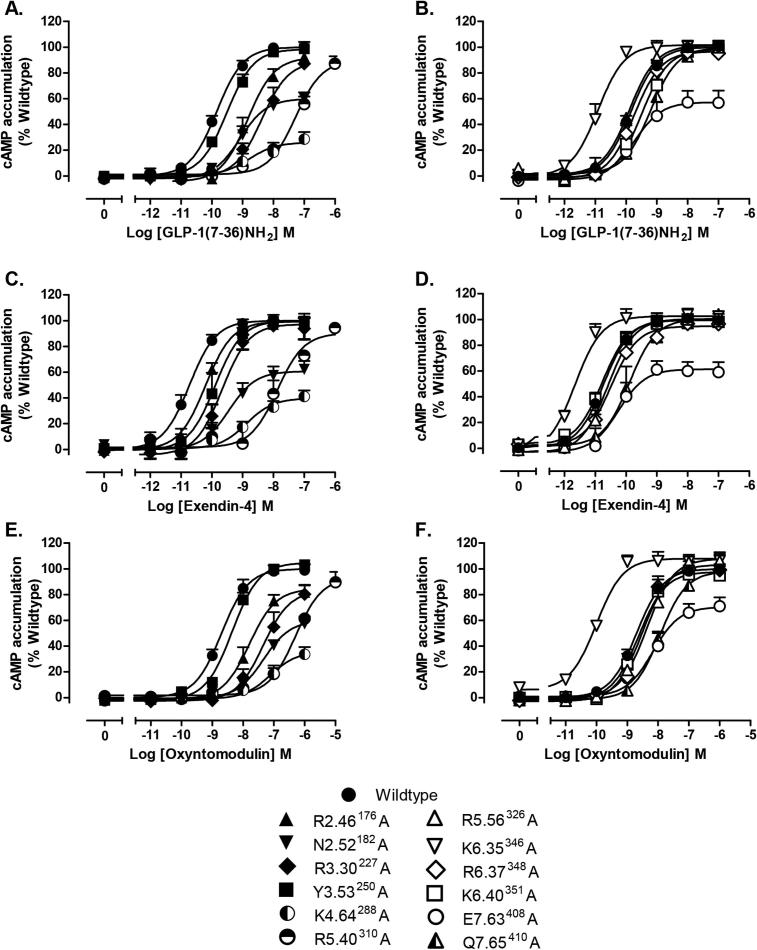
cAMP concentration–response curves for polar TM boundary Ala mutants. Concentration–response curves for cAMP accumulation of wildtype and mutant receptors stimulated by GLP-1 (A, B), exendin-4 (C, D) or oxyntomodulin (E, F) in CHOFlpIn cells stably expressing wildtype or mutant receptors. Data are normalised to the response elicited by the wildtype receptor and analysed with an operational model agonism. All values are mean ± S.E.M of four to six independent experiments, conducted in duplicate.

**Fig. 3:**
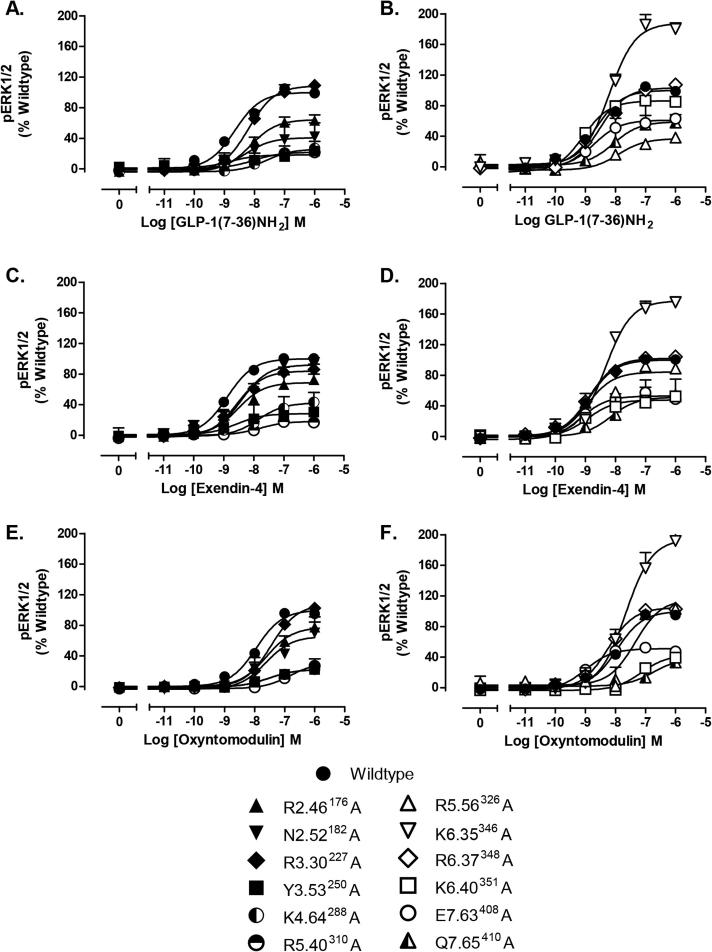
pERK1/2 concentration–response curves for polar TM boundary Ala mutants. Concentration–response curves or pERK of wildtype and mutant receptors stimulated by GLP-1 (A, B), exendin-4 (C, D) or oxyntomodulin (E, F) in CHOFlpIn cells stably expressing wildtype or mutant receptors. Data are normalised to the response elicited by the wildtype and analysed with an operational model agonism. All values are mean ± S.E.M of four to six independent experiments, conducted in duplicate.

**Fig. 4:**
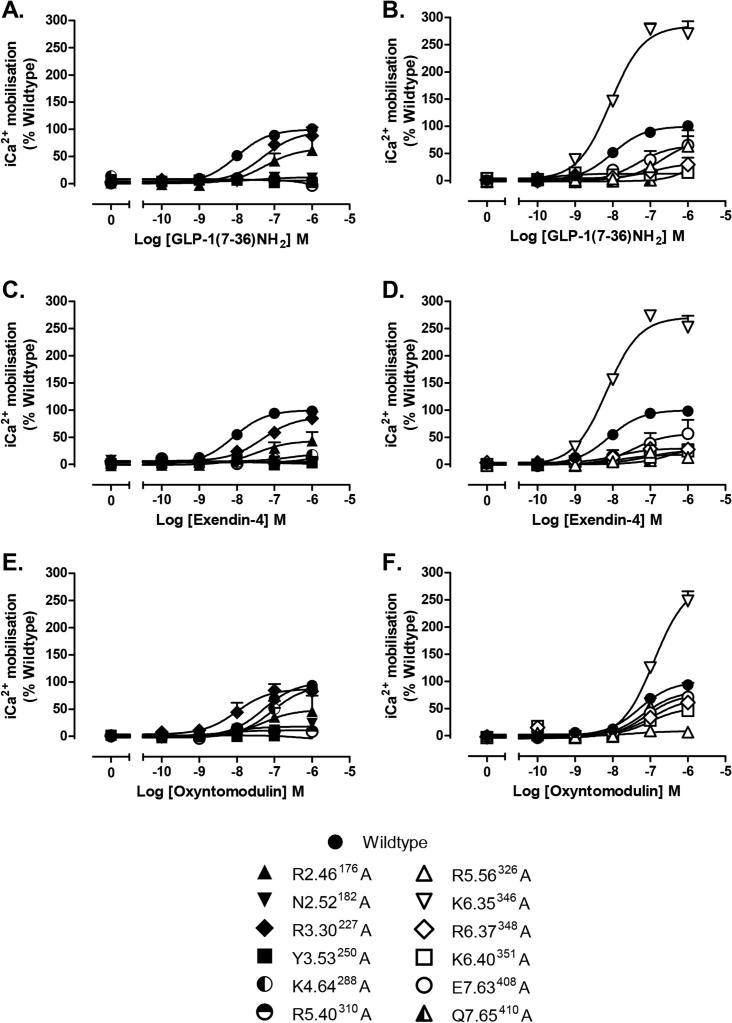
_i_Ca^2+^ mobilisation concentration–response curves for polar TM boundary Ala mutants. Concentration–response curves or _i_Ca^2+^ mobilisation of wildtype and mutant receptors stimulated by GLP-1 (A, B), exendin-4 (C, D) or oxyntomodulin (E, F) in CHOFlpIn cells stably expressing wildtype or mutant receptors. Data are normalised to the response elicited by the wildtype and analysed with an operational model agonism. All values are mean ± S.E.M of four to six independent experiments, conducted in duplicate.

**Fig. 5:**
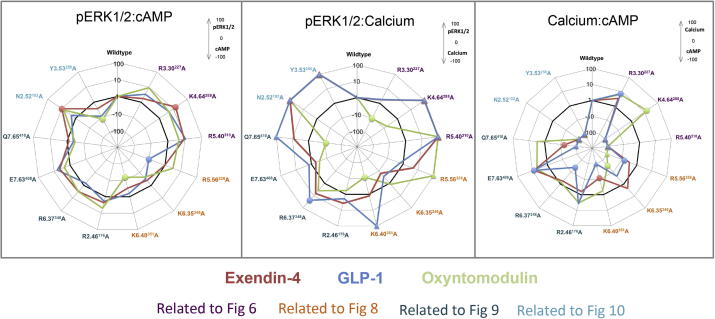
Effect of mutations on agonist bias of GLP-1R signalling pathways. Radial plots of agonist bias factors (ΔΔτ/K_A_, the ratio of the transduction coefficient for one pathway vs another, each normalised to the values determined for the wildtype receptor) derived from an operational model of agonism (see “Section 2”) plotted for each receptor variant. Values greater than 1 denote bias towards pathway 1, and values less than 1 denote bias towards pathway 2 relative to signalling at the wildtype receptor. Left, pERK1/2 (pathway 1) vs cAMP (pathway 2); middle, pERK1/2 (pathway 1) vs _i_Ca^2+^ mobilisation (pathway 2); right, _i_Ca^2+^ mobilisation (pathway 1) vs cAMP (pathway 2). All plots show the bias factors for the mutant receptors relative to the wildtype receptor for GLP-1 (blue), exendin-4 (salmon) and oxyntomodulin (green). Data points plotted as circles indicate statistically significant bias relative to the wildtype receptor (WT highlighted by the black reference line), whereas data plotted as triangles (at a value of −100 or 100) indicate that no significant signal could be detected for a particular pathway and therefor a bias factor could not be calculated. These values at −100 indicate no signalling in pathway 1 (therefore implied bias towards pathway 2), whereas +100 indicates no signalling in pathway 2 (therefore implied bias towards pathway 1). The residues are highlighted in the colour relevant to the clustering (and relevant figure) in which they are discussed in the results section.

Activation/strength of coupling to three cellular signalling cascades (cAMP production, ERK1/2 phosphorylation (pERK1/2) and intracellular calcium mobilisation (_i_Ca^2+^)) was evaluated through the generation of concentration–response curves for all receptors with each peptide agonist (Fig. 2, Fig. 3, Fig. 4). In most cases, mutations that resulted in changes in cell surface expression and/or affinity also produced significant changes on EC_50_ and/or E_max_ values (Table 2). A direct measure of efficacy via calculation of Log τ_c_ values allows for direct comparison of receptor activation of individual intracellular signalling pathways at the different receptor mutants compared to the wildtype receptor, independently of their ligand affinity and cell surface expression. These were determined by analysing all concentration–response curves using an operational model of agonism to determine relative signalling efficacy estimates (log τ values) that were corrected to different receptor expression levels by normalisation to what they would be if the mutant receptor were expressed at the same level as the wildtype (log τ_c_ values, Table 3). Cell surface expression data obtained from antibody binding were used for this correction instead of the Bmax from ligand binding studies, as one mutant showed no detectable radioligand binding, however correction with Bmax yielded similar efficacy values (data not shown). In addition, functional affinities (Log K_A_) that describe the affinity of the receptor when coupled to a given signalling pathway were also derived from the operational analysis (Table 4). The assessment of multiple signalling pathways also provided the ability to measure the signal bias of mutant receptors relative to the wildtype to obtain a quantitative measure of the relative bias between two pathways (Table 5, Fig. 5).

To aid in interpretation of the experimental data, we used our two published GLP-1R models [64]; an inactive apo model of the TM bundle only and a GLP-1R:GLP-1:G_αs_ complex that was generated using multiple structural templates (Fig. 1C–D). The combined results from expression, affinity and efficacy data (derived from the concentration–response curves) are presented in detail in the context of the predicted locations of mutated residues within these molecular models, clustering those located close in 3D space.

### 3.1 Three conserved positively charged residues located at the extracellular ends of TM helices 3, 4 and 5 are essential for high affinity agonist binding and conformational transitions linked to pleiotropic effector coupling through stabilisation of ECL2

**Fig. 6:**
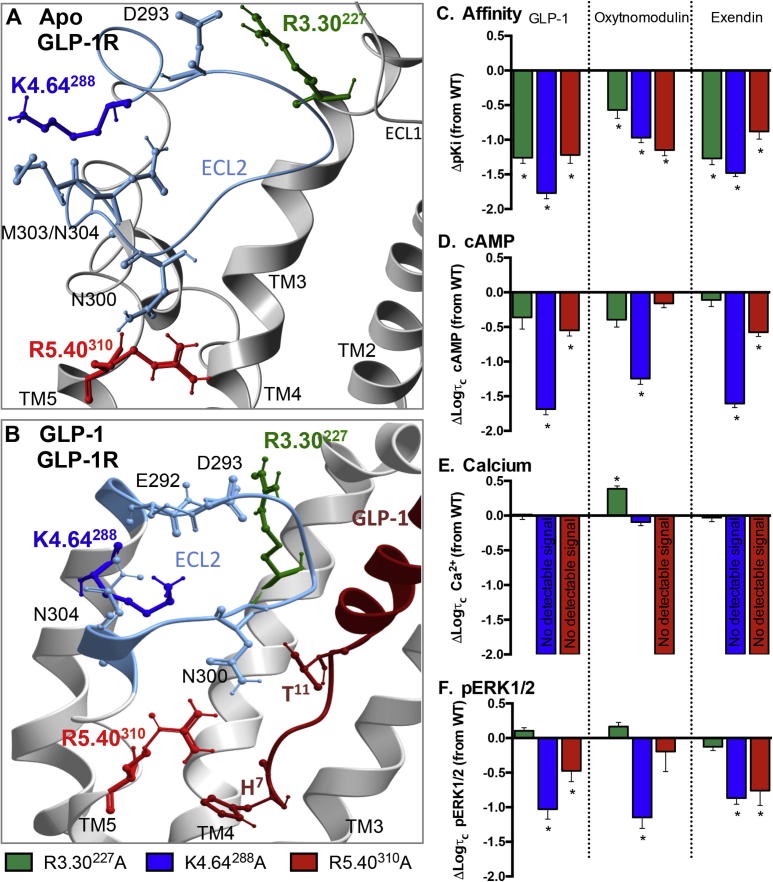
Mutation of positively charged residues predicted to interact with ECL2 impairs agonist affinity and alters receptor signalling in a pathway dependent manner. (A) Tops of TMs 2, 3, 4 and 5 of the apo GLP-1R TM bundle highlighting interactions between charged residues R3.30^227^, K4.64^288^, R5.40^310^ and residues located within ECL2 (R3.30^227^-D293, K4.64^288^-N304, R5.40^310^-N300). (B) TMs 2, 3, 4 and 5 of the GLP-1 docked activated GLP-1R TM bundle highlighting interactions between charged residues R3.30^227^, K4.64^288^, R5.40^310^ and residues located within ECL2 (R3.30^227^-D293, K4.64^288^- E292/N304, R5.40^310^-N300). Also shown is the GLP-1 peptide (dark red) with T^11^ that interacts directly with N300 located within ECL2. H^7^ of GLP-1 is also highlighted residing close to R5.40^310^. (C) Differences in equilibrium binding affinity (pKi) of mutant receptors relative to wildtype for GLP-1, oxyntomodulin and exendin-4. (D–F) Differences in the coupling efficiency (log τ_c_) of GLP-1, exendin-4 and oxyntomodulin to three signalling pathways (cAMP production (D), pERK1/2 (E) and _i_Ca^2+^ mobilisation (F)) at individual mutants compared to the wildtype receptor. These log τ_c_ were calculated from concentration–response curves presented in Fig. 2, Fig. 3, Fig. 4, and corrected for cell surface expression as measured by antibody labelling recorded in Table 1. Statistical significance of changes in affinity or coupling efficacy in comparison with wildtype were determined by one-way analysis of variance and Dunnett’s post test, and values are indicated with an asterisk (^∗^, p < 0.05). All values are ± S.E.M of four to six independent experiments, conducted in duplicate.

**Fig. 7:**
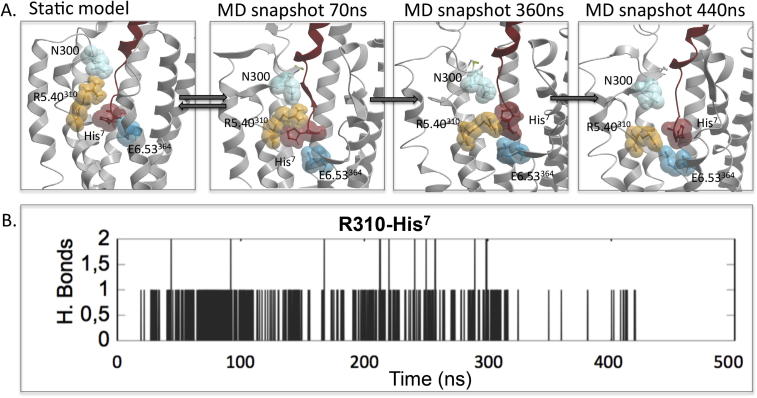
R5.40^310^ forms transient interactions with His^7^ of GLP-1. Molecular dynamics simulation was performed for a total of 500 ns commencing with the final model of the GLP-1 bound GLP-1R. (A) Interactions are identified between R5.40^310^ and both N300 and His^7^ throughout the first half of the simulation. However towards the end of the simulation the interactions with both N300 and His^7^ are lost and R5.40^310^ forms a stable interaction with E6.53^364^. (B) Hydrogen bonds formed between R5.40^310^-His^7^ during the 500 ns simulation. Hydrogen bonds were defined with the donor–acceptor distance < 3.0 Å and an angle cutoff of 20°.

Three highly conserved positively charged residues, R3.30^227^, K4.64^288^ and R5.40^310^, located close to the extracellular surface of the GLP-1R are predicted to form direct interactions with residues in ECL2 in the apo and peptide bound models (Fig. 6). R3.30^227^ is predicted to interact within the proximal region of ECL2 near to the top of TM4 in both the apo model and the GLP-1 bound model (Fig. 6). K4.64^288^ forms interactions at the opposite end of ECL2, close to the top of TM5 in the apo receptor and forms multiple interactions with ECL2 in the GLP-1 peptide bound model. In both inactive and active models, R5.40^310^ resides close to N300 that is also predicted to form a direct interaction with GLP-1. R5.40^310^ also resides close to His^7^ of GLP-1 in the active model where it may form a direct interaction (Fig. 6). MD simulations performed on this static GLP-1 bound active model revealed that R5.40^310^ forms transient hydrogen bond interactions with both N300 in ECL2 and His^7^ of GLP-1 in the first 360 ns of the MD simulation, however both of these interactions are lost towards the end of the simulation with R5.40^310^ forming a direct interaction with E6.53^364^ in TM6 (Fig. 7).

Mutation of R5.40^310^ (R5.40^310^A) resulted in a receptor that was very poorly expressed at the cell surface (<40% of wildtype), whereas R3.30^227^A and K4.64^288^A were expressed at a similar level to the wildtype receptor (Table 1). All three mutant receptors displayed a marked loss in affinity for peptide agonists (Fig. 6, Table 1). This was greater for GLP-1 and exendin-4 at R3.30^277^A (18–19-fold) and K4.64^288^A (59- and 30-fold, respectively), compared to oxyntomodulin where a 4- and 9-fold loss of affinity was observed, respectively. R5.40^310^A displayed a similar reduction in affinity for all three agonists (8–17-fold). The binding of the antagonist, exendin-4(9–39), was not altered at K4.64^288^A or R5.40^310^A compared to wildtype, whereas a small, yet significant increase in affinity was measured for R3.30^227^A (Table 1).

After correction for changes in expression, R3.30^227^A showed similar efficacy for generation of cAMP production and pERK1/2 relative to wildtype for the three peptides (Fig. 6, Table 3). However, there was a small, yet significant increase in efficacy for _i_Ca^2+^ for oxyntomodulin that was not observed with the other two peptide agonists. For R5.40^310^A, a small reduction in cAMP efficacy was observed for GLP-1 and exendin-4, but not oxyntomodulin. In addition, pERK1/2 efficacy was also slightly reduced for exendin-4 and GLP-1 (3–5-fold), but not for oxyntomodulin (Fig. 6, Table 3). In contrast, no detectable _i_Ca^2+^ was evident for any peptide at R5.40^310^A. K4.64^288^A impaired cAMP efficacy for all three peptides, but this was greater for GLP-1 and exendin-4 (42–50-fold) compared to oxyntomodulin (18-fold). In addition, there was no detectable calcium response with GLP-1 and exendin-4, although the oxyntomodulin efficacy for this pathway was unaltered. In contrast, all three ligands displayed a similar reduction in pERK1/2 efficacy (7–14-fold) (Fig. 6, Table 3).

Calculation of bias factors revealed that R5.40^310^ did not significantly alter the ability of the receptor to sample between distinct conformations for activation of pERK1/2 and cAMP. Bias could not be calculated relative to _i_Ca^2+^, as there was no detectable response for this pathway (Fig. 5, Table 5). K4.64^288^A biased the receptor towards _i_Ca^2+^ over cAMP and pERK1/2 when activated by oxyntomodulin and for exendin-4 towards pERK1/2 relative to cAMP (Fig. 5, Table 5). R3.30^227^ significantly biased GLP-1 towards _i_Ca^2+^ over cAMP, with a similar trend for oxyntomodulin and exendin-4 (Fig. 5, Table 5). This trend may not have been predicted from efficacy values alone as, unlike the majority of mutants assessed in this study, the functional K_A_ values predicted from operational modelling were also altered differentially in the distinct pathways (Table 4). The functional K_A_ linked to cAMP accumulation tracked with the loss of affinity, however in _i_Ca^2+^, little reduction in the functional K_A_ was observed compared to the wildtype receptor.

### 3.2 Three conserved positively charged residues residing near the intracellular ends of TMs 5 and 6 contribute to conformational transitions upon receptor activation

**Fig. 8:**
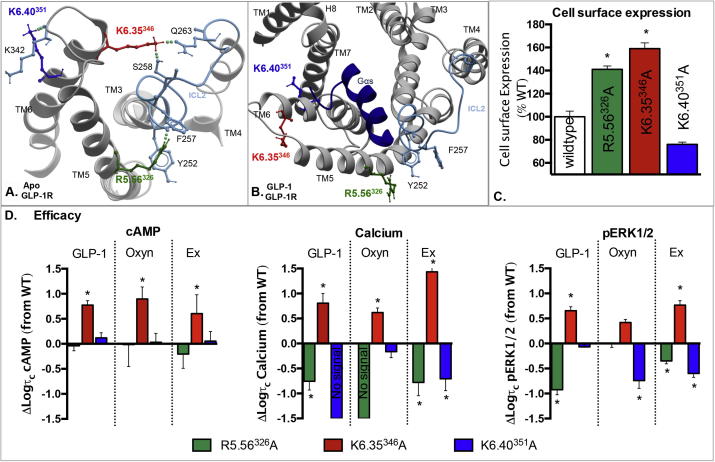
Mutation of positively charged residues predicted to interact with ICL2 and/or the lipid bilayer alters cell surface expression and receptor signalling in a pathway dependent manner. (A) TMs 3, 4, 5 and 6 of the apo GLP-1R TM bundle as viewed from the cytoplasmic face, highlighting interactions between charged residues R5.56^326^ and K6.35^346^ with residues in ICL2. K6.40^351^ is also shown where it points away from the bundle, interacting with the backbone of ICL3 and potentially interacting with lipid head groups. (B) The activated GLP-1R TM bundle as viewed from the intracellular face with a G_αs_ peptide fragment docked at the cytoplasmic face. The lipid facing location of R5.56^326^, K6.35^346^ and K6.40^351^ are highlighted. Of particular note, interactions of K6.35^346^ with ICL2 are broken to accommodate opening up of the TM bundle and G protein interaction. R5.56^326^ interactions with the backbone of ICL2 are also broken although R5.56^326^ maintains within H bond proximity to Y252. (C) Cell surface expression of mutations R5.56^326^A, K6.35^346^A and K6.40^351^A relative to the wildtype receptor as assessed by antibody binding to the N-terminal c-myc epitope tag. (D) Differences in the coupling efficiency (log τ_c_) of GLP-1, exendin-4 and oxyntomodulin to three signalling pathways (cAMP production (left), _i_Ca^2+^ mobilisation (middle), and pERK1/2 (right)) for R5.56^326^A, K6.35^346^A and K6.40^351^A compared to the wildtype receptor. These log τ_c_ were calculated from concentration–response curves presented in Fig. 2, Fig. 3, Fig. 4, and corrected for cell surface expression as measured by antibody labelling recorded in Table 1. Statistical significance of changes in cell surface expression or coupling efficacy in comparison with wildtype were determined by one-way analysis of variance and Dunnett’s post test, and values are indicated with an asterisk (^∗^, p < 0.05). All values are ± S.E.M of four to six independent experiments, conducted in duplicate.

R5.56^326^ and K6.35^346^ reside towards the intracellular side of TMs 5 and 6, respectively. In the inactive apo model, both of these residues are predicted to hydrogen bond to regions in ICL2 that may be required to stabilise ground state receptor interactions. Interestingly, alanine mutation of either of these residues increased cell surface expression (Fig. 8, Table 1). For K6.35^346^A, this was detectable by both antibody labelling (175% of wildtype) and whole cell binding (159% of wildtype). While increased expression was detectable at R5.56^326^A using antibody labelling (112% wildtype), there was significantly enhanced expression when calculating Bmax values from radioligand binding (141% of wildtype) (Table 1).

In our active, peptide bound molecular model R5.56^326^ and K6.35^346^ are predicted to undergo a reorientation compared to the apo model, with both residues pointing away from the bundle (Fig. 8). An additional charged residue, K6.40^351^ in TM6 is also located in an outward orientation relative to the bundle that is in a distinct orientation in the active model relative to the apo (Fig. 8).

While mutation of R5.56^326^ to alanine did not alter affinity of either of the peptide agonists or the antagonist exendin-4(9–39), K6.35^346^A and K6.40^351^A both had small, yet significant selective effects on ligand affinity (Table 1). K6.35^346^A selectively enhanced GLP-1 and exendin-4 affinity, with oxyntomodulin displaying a similar trend, however no effect was observed on the affinity of the antagonist. In contrast, K6.40^351^A did not alter the affinity of the peptide agonists, but showed reduced affinity for exendin-4(9–39) compared to the wildtype receptor (Table 1).

K6.35^346^A enhanced the efficacy of all three agonists for the three signalling pathways, although this did not reach statistical significance for oxyntomodulin in pERK1/2 (Fig. 8, Table 3). While GLP-1 and oxyntomodulin displayed a similar fold increase in efficacy for calcium signalling (5–6-fold), there was a larger enhancement for exendin-4 at this mutant (26-fold) (Fig. 8, Table 2).

Neither R5.56^326^A nor K6.40^351^A altered cAMP efficacy of any ligand, but both had ligand-selective negative effects on pERK1/2. R5.56^326^A reduced the efficacy of GLP-1 (8-fold) and to a lesser extent exendin-4, with no effect on oxyntomodulin. In contrast, K6.40^351^A reduced the efficacy of oxyntomodulin and exendin-4, with no effect on GLP-1. R5.56^326^A and K6.40^351^A also heavily impaired _i_Ca^2+^ when activated by GLP-1 and exendin-4, whereas oxyntomodulin-mediated _i_Ca^2+^ was impaired only at R5.56^326^A (Fig. 8, Table 3).

The ability of these mutations to selectively alter efficacy of distinct pathways and/or ligands resulted in different bias profiles of these mutant receptors relative to the wildtype (Table 5, Fig. 5). K6.35^346^A altered the coupling preference induced by oxyntomodulin, such that the receptor was even more strongly biased towards cAMP relative to _i_Ca^2+^ than wildtype, with a similar trend also seen for GLP-1 (Table 5, Fig. 5). R5.56^326^A biased GLP-1 signalling towards cAMP relative to _i_Ca^2+^ and pERK1/2. Oxyntomodulin did not signal to _i_Ca^2+^ at this mutant and therefore may be biased towards pERK1/2 and cAMP over _i_Ca^2+^ (Fig. 5, Table 5). Exendin-4 showed no significant change from wildtype at R5.56^326^A. K6.40^351^A was biased away from _i_Ca^2+^ towards both cAMP and pERK1/2 when activated by GLP-1. Exendin-4 signaling also showed a significant bias for cAMP relative to _i_Ca^2+^. In contrast, oxyntomodulin biased the signaling away from pERK1/2 relative to cAMP and _i_Ca^2+^ at this receptor in comparison to the wildtype (Fig. 5, Table 5).

### 3.3 A hydrogen bond network at the intracellular face stabilises the apo-GLP-1R and plays a role in controlling conformational transitions linked to biased signalling

**Fig. 9:**
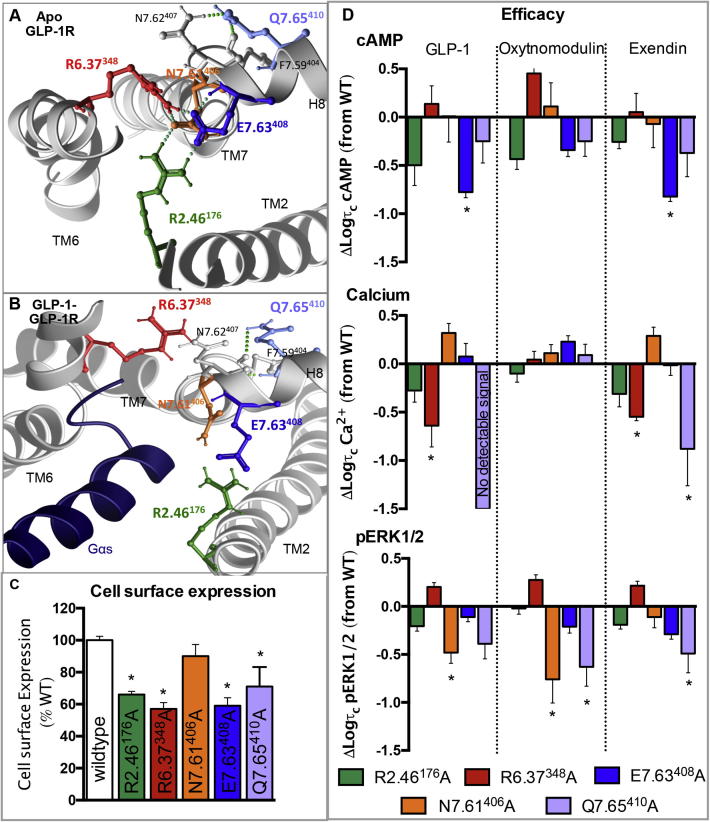
Effects on mutation of residues located in the hydrogen bonding network located between TMs 2, 6 and 7 at the cytoplasmic face. (A) TMs 2, 6, 7 and helix 8 (H8) of the apo GLP-1R TM bundle as viewed from the cytoplasmic face, highlighting an extensive hydrogen bond network between R2.46^176^, R6.37^348^, N7.61^406^ and E7.63^408^. Q7.65^410^ at the start of H8 is also shown where it forms hydrogen binds with the side chain of N7.61^407^ and the backbone of TM7 at F7.59^404^. (B) TMs 2, 6, 7 and H8 of the GLP-1 docked GLP-1R TM bundle as viewed from the cytoplasmic face with the G_αs_ peptide fragment indicating the extensive hydrogen bond network between R2.46^176^, R6.37^348^, N7.61^406^ and E7.63^408^ is broken in the activated receptor. Q7.65^410^ at the start of H8 is also shown where it still maintains a backbone interaction with F7.59^404^. (C) Cell surface expression of mutations R2.46^176^A, R6.37^348^A, N7.61^406^A, E7.63^408^A and Q7.65^410^A relative to the wildtype receptor (as assessed by antibody binding to the N-terminal c-myc epitope tag). (D) Differences in the coupling efficiency (log τ_c_) of GLP-1, exendin-4 and oxyntomodulin to three signalling pathways (cAMP production (top), _i_Ca^2+^ mobilisation (middle), and pERK1/2 (bottom)) for R2.46^176^A, R6.37^348^A, N7.61^406^A, E7.63^408^A and Q7.65^410^A compared to the wildtype receptor. These log τ_c_ were calculated from concentration–response curves presented in Fig. 2, Fig. 3, Fig. 4, and corrected for cell surface expression as measured by antibody labelling recorded in Table 1. Statistical significance of changes in cell surface expression or coupling efficacy in comparison with wildtype were determined by one-way analysis of variance and Dunnett’s post test, and values are indicated with an asterisk (^∗^, p < 0.05). All values are ±S.E.M of four to six independent experiments, conducted in duplicate.

Molecular modelling of the GLP-1R revealed a network of residues residing at the intracellular face of the receptor involving residues in TM2 (R2.46^176^), TM6 (R6.37^348^) and TM7 (N7.61^406^ and E7.63^408^). These are predicted to form an extensive hydrogen bond network in the ground state apo model (Fig. 9) that is disrupted in the active state model. We have previously reported the effects of alanine mutation of N7.61^406^ that demonstrated little effect on receptor expression, ligand binding, cAMP formation or _i_Ca^2+^ ([66], Fig. 9). However, there were small, yet significant reductions in the ability of this mutant to promote pERK1/2 when activated by GLP-1 and oxyntomodulin, but not exendin-4 (Fig. 9).

Mutation of R2.46^176^, R6.37^348^ or E7.63^408^ to alanine each resulted in a significant loss of cell surface expression (Fig. 9, Table 1). Interestingly, each mutation reduced this expression to a similar extent (57–66% of wildtype), supporting the role of these residues in a combined network. Despite this, relatively subtle effects were observed on other aspects of receptor function. All three mutants maintained the ability to bind the three agonists and the antagonist, albeit that a small yet significant reduction (4-fold) in exendin-4 affinity was observed for E7.63^408^A (Table 1). In addition, subtle changes to receptor bias occurred that did not always affect all three peptide ligands equally (Fig. 9, Table 3, Table 5). E7.63^408^A reduced cAMP signalling by all peptides, although this did not reach significance for oxyntomodulin (Fig. 9, Table 3). This resulted in E7.63^408^A being biased towards _i_Ca^2+^ relative to cAMP for all ligands, but this only reached significance for GLP-1 (Fig. 5, Table 5). R6.37^348^A selectively altered effector signalling, reducing _i_Ca^2+^ for GLP-1 and exendin-4, but not oxyntomodulin (Fig. 9, Table 3). This resulted in a statistically significant switch in the receptor bias when activated by GLP-1, such that it more readily activated effector coupling linked to pERK1/2 and cAMP compared to _i_Ca^2+^ (Table 5, Fig. 5). R2.46^176^A had no significant effect on efficacy relative to wildtype.

### 3.4 A conserved polar residue in H8 is selectively important for GLP-1 mediated signalling, with little impact on exendin-4 and oxyntomodulin

Q7.65^410^A was assessed as part of this study as it is highly conserved in class B GPCRs, but it is not located with the TM bundle, rather at the start of the predicted helix 8 (H8) at the bottom of TM7. In our apo model Q7.65^410^ is predicted to form a direct hydrogen bond with the backbone of TM7 (F7.59^404^) and with the side chain of N7.62^407^ and therefore may stabilise the hinge region between TM7 and H8 (Fig. 9). In the active model the interaction with the backbone of TM7 is maintained, but the interaction with N7.62^407^ is lost due to a reorientation of the bottom of TM7 upon activation where N7.62^407^ then resides close to the G_αs_ fragment (Fig. 9). While mutation of Q7.65^410^ slightly reduced cell surface expression, it had selective effects on GLP-1R efficacy, with no significant effect on affinity of any ligand (Table 1). GLP-1 and exendin-4 mediated cAMP formation and pERK1/2 were also unaffected, however no _i_Ca^2+^ could be detected when activated by GLP-1 and there was also reduced exendin-4 efficacy for this pathway (Fig. 9, Table 1, Table 3). This resulted in a significant bias of this mutant receptor relative to the wildtype towards cAMP formation compared to _i_Ca^2+^ for exendin-4, and implies a similar bias for GLP-1 (Fig. 5, Table 5). For oxyntomodulin a different profile was observed; this ligand displayed reduced efficacy for pERK1/2 with no effect on _i_Ca^2+^ or cAMP resulting in a significant bias of Q7.65^410^A towards _i_Ca^2+^ relative to pERK1/2 compared to the wildtype receptor (Fig. 5, Fig. 9, Table 3, Table 5).

### 3.5 N2.52^182^ and Y3.53^250^ stabilise interactions between TMs 2, 3 and 4 important for GLP-1R stability and controlling conformational transitions linked to specific activation of individual signalling pathways

**Fig. 10:**
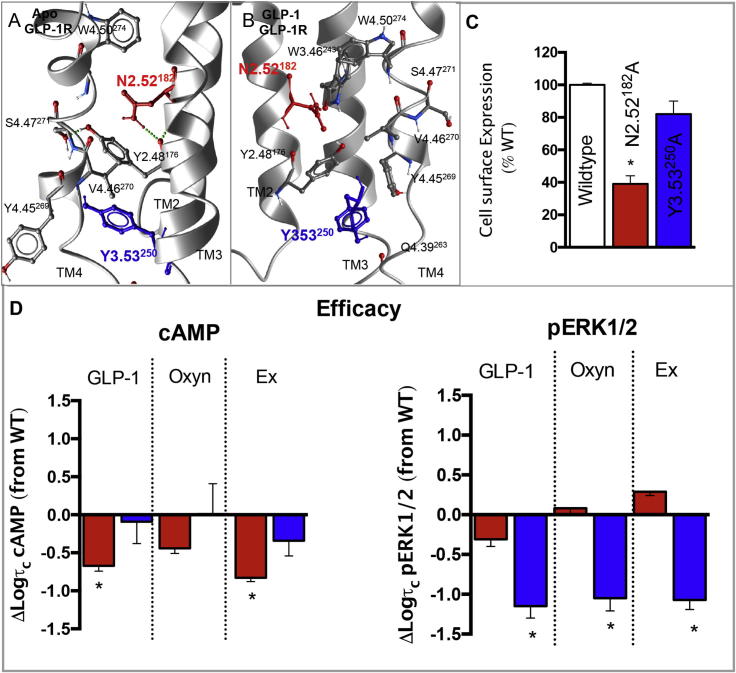
Effects on mutation of residues located TMs 2 and 3 that are predicted to intact with TM4. TMs 2, 3 and 4 of the apo GLP-1R TM bundle (A) and the activated GLP-1:GLP-1:G_αs_ peptide fragment (B) highlighting N2.52^182^ (red) and Y3.53^250^ (blue) and interacting residues within TM4 and TM3 (in the active model). (C) Cell surface expression of mutations N2.52^182^A and Y3.53^250^A relative to the wildtype receptor (as assessed by antibody binding to the N-terminal c-myc epitope tag). (D) Differences in the coupling efficiency (log τ_c_) of GLP-1, exendin-4 and oxyntomodulin to cAMP production (left) and pERK1/2 right) for N2.52^182^A and Y3.53^250^A compared to the wildtype receptor. These log τ_c_ were calculated from concentration–response curves presented in Fig. 2, Fig. 3, Fig. 4, and corrected for cell surface expression presented in (C). There was no detectable signalling for either mutant in calcium mobilisation for any of the three peptides. Statistical significance of changes in cell surface expression or coupling efficacy in comparison with wildtype were determined by one-way analysis of variance and Dunnett’s post test, and values are indicated with an asterisk (^∗^, p < 0.05). All values are ±S.E.M of four to six independent experiments, conducted in duplicate. (For interpretation of the references to colour in this figure legend, the reader is referred to the web version of this article.)

N2.52^182^ and Y3.53^250^ located in TMs 2 and 3, respectively, are predicted to form interactions with residues V4.46^270^ (and potentially W4.50^274^) and the backbone of Y4.45^269^, respectively, in the apo receptor, all located in TM4. Our GLP-1 bound active receptor model suggests a reordering of TM2 relative to TM3 and TM4 upon receptor activation resulting in formation of new interactions by the side chain of N2.52^182^. In the active state, while this residue remains close to TM4, it also interacts with Y2.48^178^ in TM2 and W3.46^243^ in TM3 (Fig. 10).

While the TM3–TM4 interaction does not appear to be important for receptor stability (as mutation of Y3.53^250^ had no effect on receptor expression), the interaction of N2.52^182^ in TM2 with TM4 residues may be important for receptor integrity as its mutation to alanine heavily impaired cell surface expression (39% of wildtype through antibody detection) (Table 1, Fig. 10). Due to this heavily impaired expression, radioligand binding could not be detected and therefore ligand affinities could not be assessed (Table 1). Following correction for the loss in cell surface expression, pERK1/2 efficacy was not significantly altered at this mutation, however cAMP production was impaired for GLP-1 and exendin-4 (5–6-fold) and no _i_Ca^2+^ could be detected for any of the three peptides (Fig. 10, Table 2, Table 3). N2.52^182^A significantly enhanced the coupling preference to pERK1/2 relative to cAMP for exendin-4 only, although a similar trend was observed with oxyntomodulin (Fig. 5, Table 5). The inability to detect an _i_Ca^2+^ signal for N2.52^182^A indicates that this receptor is likely biased towards cAMP and pERK relative to _i_Ca^2+^ for all ligands (Fig. 5, Table 5).

While mutation of Y3.53^250^ had little effect on receptor expression, agonist affinity or cAMP formation, pERK1/2 was impaired (around 10-fold) and there was no detectable _i_Ca^2+^ when activated by all three agonist peptides (Fig. 10, Table 1, Table 2, Table 3). Despite this, only oxyntomodulin displayed significantly altered bias with bias towards cAMP production relative to pERK1/2, but as there was no detectable _i_Ca^2+^ response for any peptide, it could be speculated that this mutation may also alter the bias of the GLP-1R away from _i_Ca^2+^, towards cAMP and pERK1/2 for all peptide agonists (Fig. 5, Table 5).

## 4 Discussion

Class B GPCRs are activated through interaction of the N-terminal region of their peptide agonists with the TM bundle of the receptor [47], [48], [6], [41]. ECL2 plays an important role in this activation process [23], [30], [63] and mutations within this domain in the GLP-1R result in impaired cAMP production and _i_Ca^2+^ with less dramatic effects on pERK1/2 [30], [31], [65]. In addition, these mutations within ECL2 altered the efficacy of the pERK1/2 biased agonist oxyntomodulin differentially to GLP-1 and exendin-4 highlighting a key role of this domain in biased agonism. Here, we reveal ligand-dependent roles in peptide affinity and activation of the GLP-1R of three highly conserved positively charged residues (R3.30^227^, K4.64^288^ and R5.40^310^) that have previously been implicated in GLP-1-mediated function (Table 6), and are predicted in our current molecular models to form stabilising interactions with ECL2. The conservation of positively charged residues at positions 3.30 and 4.64 in all Class B GPCRs and the negative effect on receptor function that is observed following mutation in multiple Class B GPCRs (Table 6) implies there may be a common role in stabilisation of ECL2 by these residues for this class of receptors. The distinct effects of mutation of R3.30^227^ and K4.64^288^ on affinity and efficacy of GLP-1 and exendin-4 relative to oxyntomodulin are particularly interesting as oxyntomodulin is a biased agonist relative to GLP-1 and exendin-4. These observations were more prominent for K4.64^288^ and mutation of the proposed interacting residues in ECL2 (E292A and N304A) also resulted in similar ligand-dependent changes [30], [31]. These data support a role for K4.64^288^ in controlling activation transition leading to biased agonism by influencing the conformation of ECL2 and its interaction with distinct agonists. A recent study also predicted a similar interaction of K4.64^288^ with ECL2, further supporting this theory [15]. Interestingly, for the calcitonin-like receptor (CLR) where a receptor activity modifying protein (RAMP) is required for function, mutation of R4.64 altered adrenomedullin function at CLR-RAMP2 or CLR-RAMP3 complexes, but not CGRP function at CLR-RAMP1 [60], [63]. This suggests that in Class B receptor-RAMP complexes, stabilisation of ECL2 by R/K4.64 may have distinct functional consequences, in addition to controlling biased agonism of ligands acting at the same receptor.

R5.40^310^, also conserved as a positive charge in many Class B GPCRs, interacts with ECL2 in our modelling, residing close to N300 that is predicted to form a direct interaction with GLP-1 (Fig. 6). R5.40^310^ and N300 are both required for high affinity binding of GLP-1, exendin-4 and oxyntomodulin, with mutations of each having similar effects on affinity and both affecting efficacy of all three peptide agonists [30], [31], therefore their proposed interaction may be important for peptide recognition. A polar residue at 5.40 is also required for function in other Class B GPCRs, particularly those in the glucagon subfamily (Table 6). In contrast to this proposed interaction of R5.40^310^ with N300, a recently published study predicted a direct interaction of R5.40^310^ with His^7^ of GLP-1 [15]. Although absent in our static active state model, these side chains are in close proximity and in MD simulations (500 ns), R5.40^310^ forms transient interactions with His^7^ of GLP-1 (Fig. 7). Interestingly, for the GLP-1R, R5.40^310^ also plays a role in controlling biased agonism, with distinct negative effects upon mutation for GLP-1 and exendin-4 relative to the biased ligand oxyntomodulin. Interestingly, towards the end of our 500 ns MD simulation on the GLP-1:GLP-1R model, transient interactions of R5.40^310^ with His^7^ of GLP-1 and with N300 in ECL2 are lost and R5.40^310^, as well as His^7^ of GLP-1 form stable interactions with E6.53^364^ (Fig. 6); part of a key, central, hydrogen bond network that is critical for controlling GLP-1R biased agonism [64], [65], [66]. The mutational effect of R5.40^310^ on GLP-1 and exendin-4 mediated signalling relative to oxyntomodulin is consistent with mutational studies on residues residing in this central hydrogen bond network [64], [65]; and suggests distinct functional requirements of R5.40^310^, in combination with the central hydrogen bond network for controlling peptide-mediated GLP-1R activation leading to biased agonism. These MD simulations with GLP-1 also suggest R5.40^310^ and N300 are key residues in guiding the N-terminus of these peptide agonists into the TM cavity for receptor activation (Fig. 7).

We have also previously reported on a key hydrogen bond network located at the cytoplasmic side of the TM bundle, between TMs 2, 3 and 6 that is essential for receptor integrity and for global activation of the GLP-1R [64], [66]. The current study reveals the importance of an additional hydrogen bond network, also at the intracellular face, formed by residues in TM2 (R2.46^176^), TM6 (R6.37^348^) and TM7 (N7.61^408^ and E7.63^408^) that is evident in the crystal structures of the GCGR and CRF_1_R [22], [49]. Differences in our apo models vs GLP-1 peptide bound models suggest a reorganisation of these intracellular networks involving a disruption of crucial contacts between TMs 3 and 6, and TMs 2 and 7 result in the TM bundle opening at the intracellular face, allowing for effector coupling. Mutation of these residues in both networks (with the exception of N7.61^406^) significantly reduced cell surface expression highlighting a role for both networks in receptor stability ([66], Fig. 9). The role of these networks are also consistent with experimental data from other Class B GPCRs where mutation of residues either induced constitutive cAMP activity, enhanced potency for cAMP production or result in poor receptor expression at the cell surface, observations that are all consistent with destabilisation of the inactive state [59], (Table 6). These combined data across Class B GPCRs, in addition to the conservation of these interactions in the two solved inactive state Class B GPCR TM crystal structures support a common role for hydrogen bond networks at the cytoplasmic face in stabilisation of the apo receptor [22], [49].

Residues within the newly reported TM2-6-7 network in the GLP-1R also have independent roles for signal transduction after being released from their ground state constraints. While we did not identify a role for R2.46^176^ in transmission of efficacy, it may play a minor role, as observed in a mutational study at the rat GLP-1R (Table 6). In contrast, we revealed distinct roles for R6.37^348^ and E7.63^408^ in directing signalling specificity. Consistent with other Class B GPCRs (Table 6), E7.63^408^ selectively couples the GLP-1R to cAMP (G_αs_). In contrast, R6.37^348^ plays a role in coupling the GLP-1R to _i_Ca^2+^ that is non-G_αs_-mediated [65], but only when the receptor was activated by GLP-1 and exendin-4. Along with R6.37^348^, K6.40^351^ forms part of a basic-X-X-basic motif (BxxB) that is highly conserved in both Class A and B GPCRs, but the effects of mutation are variable depending on the receptor being studied. Evidence suggest residues in this motif play only minor roles in G_αs_/cAMP efficacy for Class B GPCRs, but are more important for IP_3_/calcium mobilisation (Table 6). This is consistent with this current study on the GLP-1R, where mutation of both basic residues had little effect on cAMP production by any peptide, but reduced the efficacy of GLP-1 and exendin-4 for _i_Ca^2+^. However, there was no alteration in oxyntomodulin efficacy, consistent with distinct receptor conformational propagation achieved by the ligand that exposes distinct side chains for effector interaction. Therefore, the BxxB motif may have distinct roles in controlling receptor conformation and effector coupling between ligands acting at the same receptor. The observed effects of mutation of R6.37^348^, K6.40^351^ and E7.63^408^ for signalling specificity could arise due to direct contacts with effector proteins or indirectly through forming interactions (either within the receptor or with lipids) that stabilise active receptor conformations required for coupling to distinct pathways. Indeed, R6.37^348^ and E7.63^408^ are in the vicinity of G_αs_ in the GLP-1 bound molecular model and therefore relatively small differences in conformational rearrangement upon binding of distinct agonists could subtly alter interactions with effector proteins giving rise to the observed changes in signal bias.

Lys and Arg residues found near the polar/a-polar interfaces can hydrogen bond to phosphate head groups and esterified oxygens of the lipid backbone, anchoring TMs in the bilayer in the optimal orientation in the membrane for receptor function [51]. From our GLP-1R models, three residues R5.56^326^, K6.35^346^ and K6.40^351^ may play such a role as our active state model places these residues pointing out towards lipid. The reorientation of these three side chains between the two models suggests that these residues may be important for controlling TM movements during activation transition. Mutation of R5.56^326^ and K6.35^346^ also increased cell surface expression, an effect that is often associated with stabilisation of the ground state conformation. Indeed, Ala mutation of an equivalent residue, Y6.35, in the CRF1R TM domain crystal structure was used to increase the thermostability of the inactive receptor protein and to aid in crystallisation [22]. R5.56^326^A also selectively impaired pERK1/2 by GLP-1 and exendin-4 and heavily impaired _i_Ca^2+^ by all ligands, consistent with stabilisation of an inactive receptor. In contrast, K6.35^346^A enhanced affinity and signalling efficacy by all ligands to all three pathways. This residue is only positively charged in the glucagon subfamily of Class B GPCRs (being a polar Tyr in most others (Fig. 1)), and therefore may play a different role in this glucagon subclass compared to the other Class B members.

TM4 is the most peripherally located TM and forms the interface for GLP-1R homodimerisation in Class B GPCRs that is important for GLP-1R signalling [18]. N2.52^182^ and Y3.53^250^ pack up against TM4 and play global roles in GLP-1R activation by peptide agonists, with both residues being crucial for _i_Ca^2+^ mobilisation, but selectively involved in cAMP (N2.52^182^) or pERK signalling (Y3.53^250^), effects that may arise due to stabilisation of the important dimerisation interface. Consistent with this, mutation of either residue had the largest impact on calcium signalling, which parallels with the greater loss of calcium signalling relative to cAMP and pERK1/2 following mutation of the TM4 dimerisation interface within the GLP-1R [18]. Molecular modelling also predicts a reordering of TM2 relative to TM3 and TM4 that may stabilise residues within TM3 in the activated receptor, a key domain for signal transduction that may also contribute to the altered signalling at these mutant receptors compared to the wildtype.

Collectively, this work expands our understanding of how peptides activate the GLP-1R receptor to promote signalling, highlighting additional key conserved Class B GPCR polar side chains within the TM domain beyond those already reported. There is now a large body of evidence from multiple Class B GPCRs that shed light on how these complex receptors are activated with conserved polar residues playing a crucial role in this process (Table 6 [68], [66], [64], [65], [59], [9]. Despite their distinct mode of ligand interaction relative to Class A GPCRs, there are some parallels in how these two classes of receptors are activated. There is now substantial evidence that ECL2 plays a major role in the binding and activation of both classes of receptors [30], [63], [11], [62]. However, conformational differences within ECL2 have been identified, even within the Class A subfamily [62], suggesting different networks of interactions are involved in stabilisation of this important domain. In addition, despite different conserved amino acids in the two subclasses, polar interactions are crucial for signal propagation, facilitating conformational TM rearrangements through the reorganisation of hydrogen bond networks in Class A and Class B GPCRs [2], [3], [42], [66], [64], [9], [59]. For Class A GPCRs, there is substantial evidence that this results in a large-scale conformational transition of TM6 relative to TM3 that requires the disruption of key polar networks at the intracellular face [46], [45]. Limited evidence supports a similar movement of TM6 relative to TM3 in Class B GPCRs [50]. This study, taken together with our previous studies [66], [64], suggest that breaking of key polar networks at the intracellular face of Class B GPCRs (TM2-TM3-TM6 and TM2-TM6-TM7), like Class A GPCRs, are crucial in this subfamily of receptors to facilitate movements within TM6 allowing for effector interaction.

Additionally, there is an increasing body of evidence from mutational studies supporting distinct modes of receptor activation by biased peptides at the GLP-1R, with this study providing additional evidence for the role of polar interaction networks in influencing how these differences may be achieved. There is also evidence that the ability of individual ligands to influence polar interactions within Class A GPCRs contributes to biased agonism [53], [70]. While our mutagenesis studies combined with GLP-1R models can be used to facilitate understanding of mechanisms for activation of Class B GPCRs and propagation of biased signalling, additional and more complex structural and biophysical analysis of this receptor, (or any Class B GPCR) are required to gain an in depth understanding of the large scale conformational movements that allow these very complex receptor-ligand systems to transmit signals from the ligand binding pocket at the extracellular face to cytoplasmic signalling molecules.

## Author contributions

*Participated in research design:* Wootten, Sexton.

*Conducted experiments:* Wootten, Reynolds, Smith, Mobarec.

*Performed data analysis:* Wootten, Sexton, Mobarec, Christopoulos.

*Wrote or contributed to writing of the manuscript:* Wootten, Sexton, Reynolds, Furness, Miller, Christopoulos.

## Conflict of interest statement

The authors have no conflicts of interest to declare.

## Figures and Tables

**Table 1:** Effects of mutation on GLP-1R peptide ligand affinities and cell surface expression. Mutant and WT GLP-1Rs were stably expressed in ChoFlpIn cells and agonist affinities determined by equilibrium competition binding using [125-I]-exendin-4(9–39). Ligand affinities were determined using a three-parameter logistic equation and values are expressed as mean ± S.E.M of four to six independent experiments, conducted in duplicate. Cell surface expression was measured by ELISA against the c-myc epitope and by saturation binding, both normalised to the wildtype receptor. All data are expressed as mean ± S.E.M of four to six independent experiments, conducted in duplicate. Differences in affinity or expression were analysed with one-way analysis of variance (compared to the wildtype receptor) and Dunnett’s post test (^*^p < 0.05). ND means data were unable to be experimentally defined.

Receptor construct		Ligand binding affinity (pKi)					Cell surface expression	
		GLP-1(7*–*36)NH_2_	Oxyntomodulin	Exendin-4	Exendin-4(9*–*39)		ELISA	Bmax
Wildtype		8.67 ± 0.05	7.26 ± 0.04	8.87 ± 0.04	8.11 ± 0.04		100 ± 1	100 ± 2
R2.46^176^A		8.40 ± 0.07	7.28 ± 0.08	8.61 ± 0.11	8.17 ± 0.08		66 ± 2^∗^	72 ± 1^∗^
N2.52^182^A		ND	ND	ND	ND		39 ± 5^∗^	ND
R3.30^227^A		7.41 ± 0.08^∗^	6.69 ± 0.12^∗^	7.60 ± 0.09^∗^	8.52 ± 0.08^∗^		95 ± 4	83 ± 2
Y3.53^250^A		8.49 ± 0.09	6.99 ± 0.24	8.68 ± 0.23	7.94 ± 0.06		82 ± 8	97 ± 4
K4.64^288^A		6.90 ± 0.08^∗^	6.29 ± 0.07^∗^	7.39 ± 0.05^∗^	8.16 ± 0.05		107 ± 3	116 ± 2
R5.40^310^A		7.45 ± 0.12^∗^	6.11 ± 0.08^∗^	7.99 ± 0.11^∗^	7.87 ± 0.14		40 ± 8^∗^	23 ± 3^∗^
R5.56^326^A		8.51 ± 0.09	7.22 ± 0.06	8.59 ± 0.07	8.09 ± 0.08		112 ± 10	141 ± 3^∗^
K6.35^346^A		9.20 ± 0.07^∗^	7.68 ± 0.05	9.34 ± 0.06^∗^	8.37 ± 0.04		175 ± 13^∗^	159 ± 5^∗^
R6.37^348^A		8.38 ± 0.08	7.21 ± 0.08	8.80 ± 0.08	7.98 ± 0.08		57 ± 4^∗^	60 ± 1^∗^
K6.40^351^A		8.39 ± 0.07	7.25 ± 0.14	8.92 ± 0.06	7.76 ± 0.08^∗^		81 ± 3	76 ± 2
E7.63^408^A		8.62 ± 0.12	7.34 ± 0.09	8.29 ± 0.11^∗^	8.12 ± 0.07		59 ± 5^∗^	45 ± 4^∗^
Q7.65^410^A		8.72 ± 0.09	7.22 ± 0.05	9.08 ± 0.09	8.39 ± 0.06		71 ± 5^∗^	78 ± 7

**Table 2:** Effects of mutation on GLP-1R peptide concentration response in cAMP, pERK1/2 and _i_Ca^2+^ mobilisation. Mutant and WT GLP-1Rs were stably expressed in ChoFlpIn cells and concentration–response curves were generated in each pathway for the three agonists. pEC_50_ and E_max_ values were determined using a three-parameter logistic equation and values are expressed as mean ± S.E.M of four to six independent experiments, conducted in duplicate. Differences in pEC_50_ or E_max_ were analysed with one-way analysis of variance (compared to the wildtype receptor) and Dunnett’s post test (^*^p < 0.05). ND means data were unable to be experimentally defined.

Signalling pathway	Receptor construct	GLP-1		Oxyntomodulin		Exendin-4	
		pEC_50_	E_max_ (% WT)	pEC_50_	E_max_ (% WT)	pEC_50_	E_max_ (% WT)
cAMP	Wildtype	9.84 ± 0.06	100 ± 2	8.70 ± 0.08	100 ± 3	10.7 ± 0.08	100 ± 2
	R2.46^176^A	8.84 ± 0.09^∗^	92 ± 4	7.80 ± 0.10^∗^	85 ± 4^∗^	10.2 ± 0.09^∗^	99 ± 3
	N2.52^182^A	9.09 ± 0.12^∗^	60 ± 3^∗^	7.37 ± 0.14^∗^	60 ± 4^∗^	9.49 ± 0.19^∗^	61 ± 4^∗^
	R3.30^227^A	8.38 ± 0.12^∗^	89 ± 5	7.35 ± 0.15^∗^	83 ± 6^∗^	9.70 ± 0.12^∗^	97 ± 1
	Y3.53^250^A	9.51 ± 0.08	98 ± 3	8.35 ± 0.06	105 ± 3	9.89 ± 0.13^∗^	100 ± 4
	K4.64^288^A	8.78 ± 0.38^∗^	26 ± 4^∗^	7.12 ± 0.23^∗^	36 ± 5^∗^	8.87 ± 0.17^∗^	40 ± 3^∗^
	R5.40^310^A	7.28 ± 0.09^∗^	89 ± 4	6.30 ± 0.08^∗^	93 ± 4	7.88 ± 0.19^∗^	90 ± 7
	R5.56^326^A	9.90 ± 0.06	101 ± 2	8.37 ± 0.07	108 ± 3	10.6 ± 0.09	100 ± 4
	K6.35^346^A	10.9 ± 0.11^∗^	102 ± 3	10.0 ± 0.08^∗^	108 ± 2	11.6 ± 0.13^∗^	103 ± 3
	R6.37^348^A	9.74 ± 0.09	97 ± 3	8.52 ± 0.09	103 ± 3	10.5 ± 0.16	95 ± 4
	K6.40^351^A	9.50 ± 0.14	98 ± 5	8.63 ± 0.07	98 ± 3	10.8 ± 0.08	99 ± 2
	E7.63^408^A	9.70 ± 0.24	57 ± 5^∗^	8.17 ± 0.16^∗^	70 ± 4^∗^	10.3 ± 0.20	61 ± 4^∗^
	Q7.65^410^A	9.21 ± 0.13^∗^	100 ± 5	7.94 ± 0.06^∗^	98 ± 2	9.89 ± 0.15^∗^	100 ± 5

pERK1/2	Wildtype	8.65 ± 0.07	100 ± 2	7.95 ± 0.05	100 ± 2	8.88 ± 0.04	100 ± 1
	R2.46^176^A	8.14 ± 0.12	64 ± 3^∗^	7.61 ± 0.14	77 ± 5	8.54 ± 0.25	69 ± 6^∗^
	N2.52^182^A	8.30 ± 0.51	41 ± 8^∗^	7.63 ± 0.36	66 ± 11^∗^	8.41 ± 0.13	92 ± 4
	R3.30^227^A	8.20 ± 0.10	109 ± 4	7.46 ± 0.07	107 ± 3	8.57 ± 0.12	84 ± 4
	Y3.53^250^A	8.80 ± 0.91	18 ± 5^∗^	7.53 ± 0.21	22 ± 2^∗^	8.58 ± 0.98	28 ± 9^∗^
	K4.64^288^A	7.61 ± 0.25	26 ± 3^∗^	7.54 ± 0.22	21 ± 3^∗^	7.85 ± 0.13	42 ± 2^∗^
	R5.40^310^A	8.03 ± 0.48	22 ± 4^∗^	6.81 ± 0.25^∗^	32 ± 5^∗^	7.80 ± 0.63	18 ± 5^∗^
	R5.56^326^A	7.91 ± 0.07	37 ± 1^∗^	7.34 ± 0.20	116 ± 4	8.95 ± 0.13	84 ± 4
	K6.35^346^A	8.20 ± 0.07	188 ± 5^∗^	7.66 ± 0.10	194 ± 9^∗^	8.34 ± 0.07	177 ± 5^∗^
	R6.37^348^A	8.51 ± 0.08	103 ± 3	8.25 ± 0.09	105 ± 4	8.89 ± 0.11	102 ± 4
	K6.40^351^A	9.08 ± 0.08	86 ± 3	7.14 ± 0.11	43 ± 3^∗^	8.92 ± 0.24	47 ± 4^∗^
	E7.63^408^A	8.71 ± 0.24	61 ± 5^∗^	8.99 ± 0.11^∗^	51 ± 2^∗^	9.14 ± 0.17	53 ± 3^∗^
	Q7.65^410^A	8.07 ± 0.43	59 ± 10^∗^	6.74 ± 0.59^∗^	39 ± 14^∗^	8.16 ± 0.49	51 ± 10^∗p[]^

_i_Ca^2+^	Wildtype	8.01 ± 0.09	100 ± 4	7.29 ± 0.11	100 ± 6	8.10 ± 0.06	100 ± 3
	R2.46^176^A	7.25 ± 0.38	65 ± 12^∗^	7.36 ± 0.56	49 ± 14^∗^	7.48 ± 0.38	44 ± 8^∗^
	N2.52^182^A	ND	ND	ND	ND	ND	ND
	R3.30^227^A	7.30 ± 0.20	96 ± 9	8.01 ± 0.22	87 ± 8	7.33 ± 0.19	87 ± 8
	Y3.53^250^A	ND	ND	ND	ND	ND	ND
	K4.64^288^A	ND	ND	7.06 ± 0.15	94 ± 8	ND	ND
	R5.40^310^A	ND	ND	ND	ND	ND	ND
	R5.56^326^A	6.69 ± 0.27	76 ± 13	ND	ND	7.84 ± 0.64	18 ± 5^∗^
	K6.35^346^A	8.06 ± 0.07	285 ± 8^∗^	6.90 ± 0.06	281 ± 10^∗^	8.16 ± 0.07	271 ± 8^∗^
	R6.37^348^A	6.90 ± 0.48	33 ± 8	6.92 ± 0.19	69 ± 9	7.86 ± 0.32	30 ± 3^∗^
	K6.40^351^A	ND	ND	7.01 ± 0.27	53 ± 11^∗^	7.10 ± 0.60	26 ± 8^∗^
	E7.63^408^A	7.34 ± 0.41	65 ± 13^∗^	7.11 ± 0.12	77 ± 5	7.46 ± 0.49	57 ± 12^∗^
	Q7.65^410^A	ND	ND^∗^	7.18 ± 0.29	82 ± 13	6.47 ± 0.58^∗^	34 ± 15^∗^

**Table 3:** Effects of mutation on GLP-1R coupling efficiency to downstream effectors, cAMP, pERK1/2 and _i_Ca^2+^ mobilisation. Mutant and WT GLP-1Rs were stably expressed in ChoFlpIn cells and concentration–response curves were generated for each construct in each pathway for the three agonists. All data were analysed with an operational model of agonism to determine log τ values that define efficacy. All log τ values were corrected to cell surface expression data from the ELISA (log τ_c_). Values are expressed as mean ± S.E.M of four to six independent experiments, conducted in duplicate. Data were analysed with one-way analysis of variance and Dunnett’s post test (^*^p < 0.05). ND means data were unable to be experimentally defined.

Receptor construct	Log Tau_c_								
	cAMP			pERK1/2			_i_Ca^2+^		
	GLP-1	Oxyntomodulin	Exendin-4	GLP-1	Oxyntomodulin	Exendin-4	GLP-1	Oxyntomodulin	Exendin-4
Wildtype	1.22 ± 0.09 (17)	0.92 ± 0.16 (8.4)	1.33 ± 0.15 (21)	−0.08 ± 0.03 (0.83)	−0.07 ± 0.03 (0.84)	−0.09 ± 0.03 (0.81)	−0.30 ± 0.04 (0.50)	−0.31 ± 0.02 (0.49)	−0.31 ± 0.03 (0.49)
R2.46^176^A	0.72 ± 0.21 (5.3)	0.49 ± 0.11 (3.1)	1.07 ± 0.07 (12)	−0.29 ± 0.05 (0.52)	−0.09 ± 0.06 (0.80)	−0.28 ± 0.05 (0.52)	−0.58 ± 0.12 (0.26)	−0.40 ± 0.08 (0.40)	−0.62 ± 0.13 (0.24)
N2.52^182^A	0.55 ± 0.07 (3.5)^∗^	0.48 ± 0.07 (3.1)	0.55 ± 0.05 (3.6)^∗^	−0.39 ± 0.09 (0.41)	0.01 ± 0.09 (1.03)	0.20 ± 0.05 (1.58)	ND	ND	ND
R3.30^227^A	0.86 ± 0.17 (7.2)	0.53 ± 0.12 (3.3)	1.22 ± 0.10 (17)	0.02 ± 0.04 (1.05)	0.09 ± 0.06 (1.23)	−0.22 ± 0.05 (0.61)	−0.29 ± 0.07 (0.52)	0.08 ± 0.04 (1.20)^∗^	−0.34 ± 0.06 (0.46)
Y3.53^250^A	1.13 ± 0.29 (13)	0.93 ± 0.40 (8.5)	0.99 ± 0.20 (10)	−1.23 ± 0.15 (0.06)^∗^	−1.12 ± 0.16 (0.08)^∗^	−1.16 ± 0.12 (0.07)^∗^	ND	ND	ND
K4.64^288^A	−0.47 ± 0.08 (0.34)^∗^	−0.32 ± 0.08 (0.48)^∗^	−0.28 ± 0.05 (0.5)^∗^	−1.11 ± 0.14 (0.08)^∗^	−1.22 ± 0.16 (0.06)^∗^	−0.96 ± 0.09 (0.11)^∗^	ND	−0.40 ± 0.05 (0.39)	ND
R5.40^310^A	0.67 ± 0.09 (4.7)^∗^	0.76 ± 0.06 (5.8)	0.75 ± 0.06 (5.6)^∗^	−0.55 ± 0.15 (0.28)	−0.27 ± 0.29 (0.54)	−0.85 ± 0.21 (0.14)^∗^	ND	ND	ND
R5.56^326^A	1.18 ± 0.10 (15)	0.91 ± 0.14 (8.0)	1.12 ± 0.13 (13)	−1.01 ± 0.10 (0.10)^∗^	−0.07 ± 0.08 (0.85)	−0.44 ± 0.06 (0.36)^∗^	−1.06 ± 0.17 (0.09)^∗^	ND	−1.09 ± 0.27 (0.08)^∗^
K6.35^346^A	1.99 ± 0.10 (98)^∗^	1.81 ± 0.24 (66)^∗^	1.93 ± 0.37 (85)^∗^	0.57 ± 0.09 (3.72)^∗^	0.35 ± 0.06 (2.21)	0.68 ± 0.09 (4.74)^∗^	0.50 ± 0.20 (3.2)^∗^	0.32 ± 0.09 (2.1)^∗^	1.12 ± 0.21 (13)^∗^
R6.37^348^A	1.36 ± 0.19 (16)	1.37 ± 0.35 (23)	1.19 ± 0.12 (16)	0.12 ± 0.04 (1.32)	0.20 ± 0.06 (1.59)	0.13 ± 0.05 (1.33)	−0.94 ± 0.21 (0.11)^∗^	−0.16 ± 0.08 (0.69)	−0.86 ± 0.04 (0.14)^∗^
K6.40^351^A	1.34 ± 0.10 (22)	0.95 ± 0.17 (9.0)	1.38 ± 0.19 (24)	−0.15 ± 0.04 (0.71)	−0.82 ± 0.16 (0.15)^∗^	−0.69 ± 0.08 (0.20)^∗^	ND	−0.47 ± 0.11 (0.34)	−1.02 ± 0.22 (0.10)^∗^
E7.63^408^A	0.44 ± 0.06 (2.8)^∗^	0.58 ± 0.07 (3.8)	0.50 ± 0.05 (3.2)^∗^	−0.19 ± 0.05 (0.64)	−0.28 ± 0.07 (0.52)	−0.38 ± 0.05 (0.42)	−0.23 ± 0.13 (0.59)	−0.07 ± 0.06 (0.85)^∗^	−0.33 ± 0.10 (0.47)
Q7.65^410^A	0.97 ± 0.10 (9.3)	0.67 ± 0.07 (4.7)	0.96 ± 0.11 (9.2)	−0.47 ± 0.07 (0.40)	−0.70 ± 0.19 (0.20)^∗^	−0.58 ± 0.09 (0.26)^∗^	ND	−0.22 ± 0.05 (0.60)	−1.19 ± 0.17 (0.07)^∗^

**Table 4:** Effects of mutation on the function K_A_ derived from operational fitting to cAMP, pERK1/2 and _i_Ca^2+^ mobilisation data. Mutant and WT GLP-1Rs were stably expressed in ChoFlpIn cells and concentration-response curves were generated for each construct in each pathway for the three agonists. All data were analysed with an operational model of agonism (Eq. 2) to determine Log K_A_ (functional affinity) values. Values are expressed as mean ± S.E.M of four to six independent experiments, conducted in duplicate. ND means data were unable to be experimentally defined.

Receptor construct	−Log K_A_								
	cAMP			pERK1/2			iCa^2+^		
	GLP-1	Oxyntomodulin	Exendin-4	GLP-1	Oxyntomodulin	Exendin-4	GLP-1	Oxyntomodulin	Exendin-4
Wildtype	8.35 ± 0.10	7.44 ± 0.09	9.24 ± 0.10	7.84 ± 0.11	7.46 ± 0.08	8.31 ± 0.07	7.32 ± 0.14	7.23 ± 0.34	7.46 ± 0.05
R2.46^176^A	8.08 ± 0.22	7.01 ± 0.10	8.95 ± 0.36	7.75 ± 0.14	7.39 ± 0.13	8.12 ± 0.09	7.26 ± 0.13	7.38 ± 0.04	7.39 ± 0.12
N2.52^182^A	8.50 ± 0.09	7.13 ± 0.13	9.16 ± 0.18	7.91 ± 0.13	7.40 ± 0.12	8.21 ± 0.12	ND	ND	ND
R3.30^227^A	7.50 ± 0.27	6.69 ± 0.22	8.13 ± 0.10	6.81 ± 0.06	6.81 ± 0.13	7.31 ± 0.05	6.79 ± 0.21	7.81 ± 0.24	6.82 ± 0.13
Y3.53^250^A	8.24 ± 0.10	7.23 ± 0.22	9.37 ± 0.10	7.70 ± 0.11	7.11 ± 0.08	8.11 ± 0.19	ND	ND	ND
K4.64^288^A	6.91 ± 0.32	6.29 ± 0.06	8.16 ± 0.23	7.13 ± 0.40	7.30 ± 0.38	7.01 ± 0.12	ND	6.90 ± 0.17	ND
R5.40^310^A	7.52 ± 0.35	6.07 ± 0.41	7.79 ± 0.30	7.91 ± 0.15	6.35 ± 0.35	7.53 ± 0.31	ND	ND	ND
R5.56^326^A	8.61 ± 0.13	7.56 ± 0.17	9.22 ± 0.13	7.83 ± 0.11	7.10 ± 0.28	8.33 ± 0.41	7.26 ± 0.17	ND	7.10 ± 0.19
K6.35^346^A	9.18 ± 0.19	7.64 ± 0.07	9.91 ± 0.11	8.32 ± 0.04	7.68 ± 0.03	8.21 ± 0.07	7.91 ± 0.15	7.27 ± 0.16	7.78 ± 0.08
R6.37^348^A	8.53 ± 0.21	7.43 ± 0.08	9.37 ± 0.10	7.73 ± 0.12	7.39 ± 0.17	8.35 ± 0.30	7.50 ± 0.14	7.01 ± 0.10	7.08 ± 0.09
K6.40^351^A	8.14 ± 0.23	7.48 ± 0.10	9.31 ± 0.09	7.80 ± 0.15	7.51 ± 0.19	8.12 ± 0.19	ND	7.40 ± 0.10	7.15 ± 0.21
E7.63^408^A	8.87 ± 0.18	7.11 ± 0.11	8.89 ± 0.18	7.81 ± 0.09	7.02 ± 0.19	8.35 ± 0.27	7.16 ± 0.21	6.91 ± 0.23	7.01 ± 0.09
Q7.65^410^A	8.01 ± 0.31	6.99 ± 0.33	9.41 ± 0.11	7.70 ± 0.18	7.33 ± 0.23	8.35 ± 0.27	ND	7.25 ± 0.15	7.10 ± 0.30

**Table 5:** Effects of GLP-1R mutation on signal pathway bias. Data were analysed using an operational model of agonism to estimate log τ_c_/K_A_ ratios. Changes in log τ_c_/K_A_ ratios with respect to WT were calculated to provide a measure of the degree of stimulus bias exhibited by mutant receptors across the three pathways relative to that of the control receptor (WT). Values are expressed as mean ± S.E.M of four to six independent experiments, conducted in duplicate. Data were analysed with one-way analysis of variance and Dunnett’s post test (^*^p < 0.05). ND indicates data unable to be experimentally defined.

	Δlog Rn relative to WT								
	pERK1/2-cAMP			ERK-_i_Ca^2+^			_i_Ca^2+^-cAMP		
	GLP-1	Oxyntomodulin	Exendin-4	GLP-1	Oxyntomodulin	Exendin-4	GLP-1	Oxyntomodulin	Exendin-4
Wildtype	0.00 ± 0.12 (1.0)	0.00 ± 0.07 (1.0)	0.00 ± 0.09 (1.0)	0.00 ± 0.11 (1.0)	0.00 ± 0.10 (1.0)	0.00 ± 0.10 (1.0)	0.00 ± 0.13 (1.0)	0.00 ± 0.15 (1.0)	0.00 ± 0.09 (1.0)
R2.46^176^A	0.29 ± 0.15 (1.9)	0.70 ± 0.15 (5.0)	0.37 ± 0.16 (2.3)	0.29 ± 0.29 (1.9)	−0.24 ± 0.33 (0.57)	0.57 ± 0.31 (3.7)	0.57 ± 0.32 (3.7)	0.52 ± 0.33 (3.3)	−0.21 ± 0.31 (0.62)
N2.52^182^A	0.31 ± 0.32 (2.0)	0.86 ± 0.21 (7.2)	0.99 ± 0.20 (9.8)^∗^	ND	ND	ND	ND	ND	ND
R3.30^227^A	0.52 ± 0.24 (3.3)	0.97 ± 0.12 (9.3)	0.25 ± 0.17 (1.8)	0.26 ± 0.17 (1.8)	−1.06 ± 0.15 (0.09)^∗^	0.24 ± 0.18 (1.7)	0.86 ± 0.26 (7.2)^∗^	0.81 ± 0.17 (6.4)	0.52 ± 0.14 (3.3)
Y3.53^250^A	−0.94 ± 0.39 (0.11)	−1.13 ± 0.36 (0.07)^∗^	−0.44 ± 0.30 (0.36)	ND	ND	ND	ND	ND	ND
K4.64^288^A	0.69 ± 0.32 (4.9)	0.68 ± 0.37 (4.8)	1.17 ± 0.12 (15)^∗^	ND	−0.80 ± 0.28 (0.16)	ND	ND	1.17 ± 0.32 (15)^∗^	ND
R5.40^310^A	1.04 ± 0.41 (11)	0.65 ± 0.33 (4.5)	0.96 ± 0.49 (9.1)	ND	ND	ND	ND	ND	ND
R5.56^326^A	−1.03 ± 0.23 (0.09)^∗^	0.49 ± 0.16 (3.1)	−0.02 ± 0.17 (0.95)	−0.19 ± 0.35 (0.64)	ND	0.67 ± 0.57 (4.7)	−0.83 ± 0.18 (0.15)^∗^	ND	−0.50 ± 0.35 (0.32)
K6.35^346^A	−0.51 ± 0.26 (0.31)	−0.65 ± 0.26 (0.22)	−0.38 ± 0.11 (0.42)	−0.56 ± 0.23 (0.34)	0.08 ± 0.24 (1.2)	−0.82 ± 0.07 (0.15)	−0.66 ± 0.13 (0.22)	−1.54 ± 0.11 (0.03)^∗^	0.37 ± 0.10 (2.3)
R6.37^348^A	−0.16 ± 0.16 (0.69)	0.49 ± 0.13 (3.1)	0.49 ± 0.14 (3.1)	1.38 ± 0.36 (24)^∗^	0.61 ± 0.24 (4.1)	0.84 ± 0.09 (6.9)	−1.40 ± 0.25 (0.04)^∗^	−0.11 ± 0.23 (0.79)	−0.25 ± 0.29 (0.56)
K6.40^351^A	−0.19 ± 0.21 (0.65)	−1.18 ± 0.28 (0.07)^∗^	−0.51 ± 0.24 (0.30)	ND	−1.07 ± 0.18 (0.09)^∗^	0.10 ± 0.24 (1.3)	ND	−0.24 ± 0.29 (0.57)	−1.08 ± 0.21 (0.08)^∗^
E7.63^408^A	0.80 ± 0.27 (6.3)	0.36 ± 0.21 (2.3)	0.64 ± 0.22 (4.4)	0.15 ± 0.33 (1.4)	−0.54 ± 0.27 (0.29)	−0.30 ± 0.29 (0.50)	0.90 ± 0.15 (8.0)^∗^	0.70 ± 0.24 (5.0)	0.88 ± 0.29 (7.7)
Q7.65^410^A	−0.37 ± 0.10 (0.43)	−0.48 ± 0.44 (0.33)	−0.10 ± 0.25 (0.79)	ND	−1.07 ± 0.48 (0.08)^∗^	0.90 ± 0.54 (7.9)	ND	0.47 ± 0.18 (3.0)	−1.24 ± 0.52 (0.06)^∗^

**Table 6:** Published information for Class B GPCRs following mutation of the conserved polar residues assessed in this study. h, human; o, opossum; r, rat. GLP-1(R); glucagon-like peptide-1 (receptor); CLR, calcitonin-like receptor; RAMP, receptor activity modifying protein; CGRP, calcitonin gene related peptide; SecR, secretin receptor; PTH-(R), parathyroid hormone (receptor); GCGR, glucagon receptor; VPAC-(R), vasoactive intestinal polypeptide (receptor); GIP(R), glucose-dependent insulinotropic peptide (receptor). CRE; cAMP response element.

Position (Class B Wootten numbering)	Mutant	Receptor	Effect compared with WT	Reference
2.46	R2.46A	hGLP-1R	Decreased GLP-1 mediated cAMP potency	[38]
	R2.46A	rGCGR	No detectable cell surface expression	[52]
	R2.46A	hCLR-RAMP 1	Reduced CGRP mediated cAMP potency.	[59]
	R2.46A	SecR	Decreased secretin mediated calcium potency but not cAMP potency	[17]
2.52	N2.52A	hCLR-RAMP1	No effect on CGRP affinity or cAMP production	[59]
	H2.52A	oPTH-1R	No effect on PTH-1 cAMP production.	[57]
3.30	R3.30A	rGLP-1R	Reduced GLP-1 mediated cAMP production	[69]
	R3.30A	hGCGR	Reduced expression and glucagon affinity	[15]
	R3.30A	rSecR	Reduced secretin-mediated cAMP production	[14]
	K3.30A	hCLR-RAMP1	No effect on CGRP mediated cAMP production	[59]
	K3.30A	hCLR-RAMP2	Reduced adrenomedullin cAMP production	[60]
	K3.30A	hCLR-RAMP3	Reduced adrenomedullin cAMP production	[60]
3.53	Y3.53A	hVPAC1R	Reduced VIP mediated cAMP production	[55]
4.64	K4.64A	rGLP-1R	Reduced GLP-1 affinity	[1]
	K4.64A	hGLP-1R	Reduced expression, GLP-1 affinity and cAMP efficacy	[15]
	K4.64L	hGCGR	Reduced glucagon affinity	[15]
	R4.64A	oPTH-1R	No effect on PTH mediated cAMP	[57]
	R4.64A	rSecR	Decreased secretin mediated cAMP potency.	[14]
	R4.64A	hCLR-RAMP1	Reduced CGRP mediated cAMP pEC_50_.	[59]
	R4.64A	hCLR-RAMP2	Reduced adrenomedullin mediated cAMP production	[60]
	R4.64A	hCLR-RAMP3	Reduced adrenomedullin mediated cAMP production	[60]
5.40	R5.40A	hGLP-1R	Reduced expression, GLP-1 affinity and GLP-1 mediated cAMP potency.	[12]
	R5.40A	hGLP-1R	Reduced expression, GLP-1 affinity and cAMP efficacy.	[15]
	R5.40A	hGCGR	Reduced expression and glucagon affinity	[15]
	R5.40A	hGIPR	Reduced GIP mediated cAMP production.	[71]
	H5.40A	hCLR-RAMP1	Reduced CGRP-mediated cAMP pEC_50_	[59]
5.56	N5.56A	hCLR-RAMP1	No effect on CGRP cAMP mediated production	[59]
6.35	Y6.35A	hVPAC1R	No effect on VIP mediated cAMP	[13]
6.37	K6.37A	hCLR-RAMP1	No effect on CGRP mediated cAMP production	[10]
	R6.37A	hVPAC1R	No effect on VIP mediated cAMP production	[13]
	R6.37A	hSecR	No effect on secretin mediated cAMP production	[8]
	R6.37G	rGLP-1R	Decreased GLP-1 affinity	[20]
	R6.37A	rGLP-1R	No effect on GLP-1 mediated cAMP production	[54]
	R6.37A	rGCGR	Enhanced glucagon mediated CRE reporter activity (potency and Emax)	[52]
	R6.37A	hVPAC2R	Reduced VIP mediated cAMP potency	[35]
	K6.37A	hCRF-1R	Increased CRF mediated cAMP potency (Gs), reduced pERK1/2 (Gi)	[44]
6.37/6.40	R6.37A/K6.40A	hSecR	Reduced secretin mediated cAMP and calcium, no effect on affinity or receptor expression	[17]
6.40	R6.40A	hCLR-RAMP1	5-fold reduction in CGRP affinity, 30-fold reduction in CGRP mediated cAMP production	[10]
	R6.40A	hVPAC1R	Reduced VIP mediated IP3 production, no effect on cAMP	[36]
	K6.40A	rGLP-1R	No effect on GLP-1 mediated cAMP production	[54]
	R6.40A	hVPAC2R	Reduced VIP mediated cAMP potency	[35]
	K6.40A	hCRF-1R	Increased urocortin mediated cAMP (Gs), reduced IP_3_ (Gq)	[44]
7.61	N7.61A	hGLP-1R	No effect on expression, affinity, cAMP or calcium mobilisation, but reduced GLP-1 and oxyntomodulin mediated pERK1/2 (not exendin-4)	[66]
	N7.61A	rGCGR	Enhanced potency in glucagon mediated CRE reporter activity assay	[52]
7.63	E7.63A	hCLR-RAMP1	Reduced CGRP-mediated cAMP potency	[59]
	E7.63A	rGCGR	Enhanced basal activity and enhanced potency in glucagon mediated CRE reporter activity assay	[52]
	E7.63 K	oPTH-1R	No effect on PTH mediated cAMP	[57]
	E7.63A	hVPAC1R	Decreased VIP mediated cAMP production	[13]
